# Overexpression of AHL proteins enhances root hair production by altering the transcription of RHD6‐downstream genes

**DOI:** 10.1002/pld3.517

**Published:** 2023-08-02

**Authors:** Qike Zeng, Li Song, Mingzhe Xia, Zai Zheng, Ziang Chen, Ximing Che, Dong Liu

**Affiliations:** ^1^ MOE Key Laboratory of Bioinformatics, Center for Plant Biology, School of Life Sciences Tsinghua University Beijing China; ^2^ State Key Laboratory of Crop Gene Exploration and Utilization in Southwest China Sichuan Agricultural University at Wenjiang Chengdu China; ^3^ Hainan Yazhou Bay Seed Laboratory Sanya China

**Keywords:** AHL proteins, *Arabidopsis thaliana*, gene transcription, HSP70, protein–protein interaction, regulatory mechanism, RHD6, root hair development

## Abstract

AT‐HOOK MOTIF NUCLEAR LOCALIZED (AHL) proteins occur in all sequenced plant species. They bind to the AT‐rich DNA sequences in chromosomes and regulate gene transcription related to diverse biological processes. However, the molecular mechanism underlying how AHL proteins regulate gene transcription is poorly understood. In this research, we used root hair production as a readout to study the function of two Arabidopsis AHL proteins, AHL17, and its closest homolog AHL28. Overexpression of *AHL17* or *AHL28* greatly enhanced root hair production by increasing the transcription of an array of genes downstream of RHD6. RHD6 is a key transcription factor that regulates root hair development. Mutation of *RHD6* completely suppressed the overproduction of root hairs by blocking the transcription of *AHL17*‐activated genes. The overexpression of *AHL17* or *AHL28*, however, neither affected the transcription of *RHD6* nor the accumulation of RHD6 protein. These two AHL proteins also did not directly interact with RHD6. Furthermore, we found that three members of the Heat Shock Protein70 family, which have been annotated as the subunits of the plant Mediator complex, could form a complex with both AHL17 and RHD6. Our research might reveal a previously unrecognized mechanism of how AHL proteins regulate gene transcription.

## 1 INTRODUCTION

AT‐HOOK MOTIF NUCLEAR LOCALIZED (AHL) is a group of proteins that exist in eukaryotes, including all sequenced plant species (Zhao et al., 2014). AHL proteins contain two conserved structural units: an AT‐hook motif located at their N‐terminus and a plant‐ and prokaryote‐conserved (PPC) domain located at their C‐terminus (Fujimoto et al., 2004). The AT‐hook motif has a conserved palindromic core sequence of Arg–Gly–Arg, which binds to the AT‐rich region in the minor groove of DNA (Aravind & Landsman, 1998; Reeves & Nissen, 1990; Strick & Laemmli, 1995). The PPC domain is involved in protein–protein interaction and nucleus targeting (Zhao et al., 2013). In higher plants, AHL proteins have been shown to regulate a variety of processes involved in growth and development, such as flower initiation (Yun et al., 2012), flower organ patterning (Ng et al., 2009), vascular tissue patterning (Zhou et al., 2013), leave senescence (Lim et al., 2007), hypocotyl growth (Zhao et al., 2013), pollen wall formation (Lou et al., 2014), gibberellin biosynthesis (Matsushita et al., 2007), axillary meristem maturation (Karami et al., 2020), and embryo development (Karami et al., 2021). The model plant *Arabidopsis thaliana* (Arabidopsis) has 29 AHL family members, which can be divided into two phylogenic clades (Figure S1A). Several Arabidopsis AHL proteins have been shown to regulate gene transcription by binding to a specific AT‐rich DNA sequence and by interacting with some transcription factors (Lee & Seo, 2017; Ng et al., 2009; Zhao et al., 2013). To date, however, it is still unknown whether nuclear proteins other than the general transcription machinery and transcription factors are involved in AHL‐mediated gene transcription in higher plants.

In this research, we used root hair production in Arabidopsis as a readout to study the working mechanism of two Arabidopsis AHL proteins, AHL17 and AHL28, in regulating gene transcription. Root hairs are tubular outgrowths of root epidermal cells (Dolan et al., 1994; Gilroy & Jones, 2000). Their formation is controlled by a complex regulatory network of gene transcription. In Arabidopsis, the fate of root hair cells is determined by a homeodomain‐type transcription factor GLABRA2 (GL2) (Di Cristina et al., 1996; Masucci & Schiefelbein, 1996; Rerie et al., 1994). After root hair cells are specified, the signaling components downstream of GL2 promote root hair initiation and elongation, which involves cell expansion. These signaling components include the following bHLH‐type transcription factors: ROOT HAIR DEFECTIVE6 (RHD6), its homologous proteins RHD6‐LIKE1 to 5 (RSL1–5) (Menand et al., 2007), and orthologs of *Lotus japonica*‐ROOTHAIRLESS1‐LIKE1 to 3 (LRL1–3) (Karas et al., 2009). Among them, RHD6 and RSL1 act immediately downstream of GL2 to control the transcription of *RSL2–5* and *LRL1–3*, in which RHD6 plays a key role (Bruex et al., 2012; Lin et al., 2015; Pires et al., 2013; Yi et al., 2010). Downstream of these transcription factors are the proteins that directly participate in cell expansion, which involves several cellular processes, such as Ca^2+^ and H^+^ transport, cytoskeleton reorganization, vesicular trafficking, ROS production, synthesis of cell wall materials, and cell wall modifications (Grierson et al., 2014; Ishida et al., 2008).

Heat Shock Protein 70s (HSP70s) are the most abundant proteins induced by high temperature and are highly conserved in both prokaryotes and eukaryotes (Nover & Scharf, 1997). They function as molecular chaperons that help proteins fold properly and that prevent proteins from forming aggregates under stress conditions (Usman et al., 2017). HSP70s are also involved in protein translation and translocation and in stabilization of the cell membrane. In Arabidopsis, the HSP70 family has 18 members, which are located in various subcellular compartments (Usman et al., 2017). The Arabidopsis mutants with knockout of multiple *HSP70* genes exhibited altered developmental phenotypes and decreased tolerance to several abiotic stresses (Leng et al., 2017).

Using combined genetic and molecular approaches, we found in the current research that overexpression of *AHL17* and *AHL28* promoted root hair production through enhancing the transcription of an array of the genes downstream of RHD6. Further investigation showed that three members of the HSP70 family might serve as the molecular links between AHL17/AHL28 and RHD6 in regulating gene transcription. Our research therefore revealed a previously unrecognized mechanism of how AHL proteins regulate gene transcription. This work also established an ideal experimental system for further studying the function of AHL proteins and may have some applications in engineering transgenic crops with high water and nutrition efficiency.

## 2 RESULTS

### 2.1 Overexpression of *AHL17* and *AHL28* enhances root hair production

**FIGURE 1:**
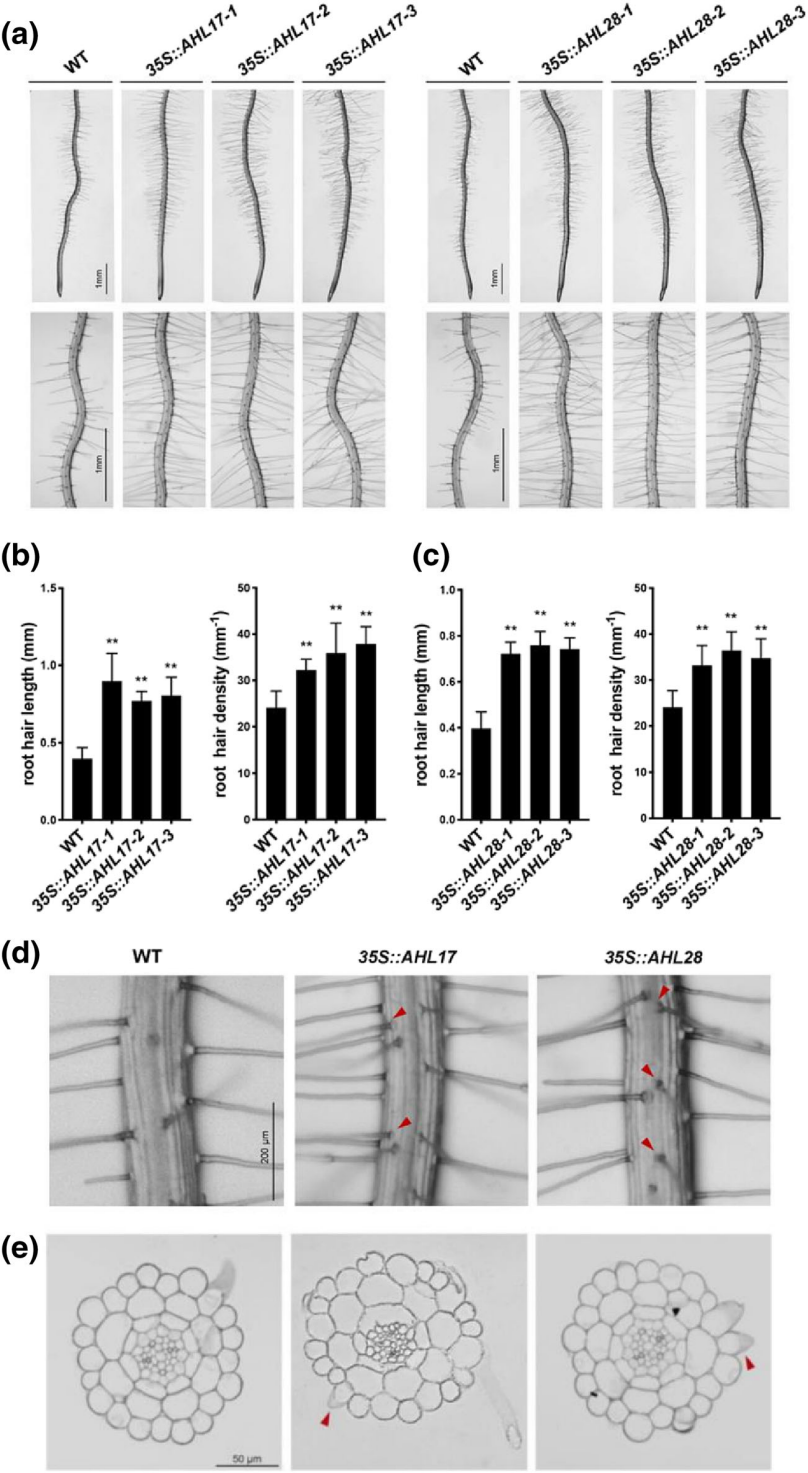
Root hair phenotypes of *AHL17*‐ and *AHL28*‐ovrexpressing lines. (a) Root hair phenotypes of 7‐day‐old seedlings of the WT, three independent *35S::AHL17* lines and *35S::AHL28* lines. (b) and (c) Root hair length and density of the WT, *35S::AHL17* lines and *35S::AHL28* lines shown in (a). Values are means ± SD of at least 15 roots for each line. Asterisks indicate a significant difference from the WT (*t*‐test, **P < .01). (d) Roots of 7‐day‐old seedlings of the WT, *35S::AHL17*, and *35S::AHL28* lines were examined under a stereomicroscope. Red arrows indicate the root hairs produced from the non‐hair position. (e) Cross‐section of 7‐day‐old seedlings of the WT, *35S::AHL17*, and *35S::AHL28* lines. Red arrows indicate root hairs produced at non‐hair positions.

We previously identified an Arabidopsis mutant, *hps5*, that has enhanced root hair production (Song et al., 2016). *hps5* carries a gain‐of‐function mutation in the ethylene receptor ERS1 and its roots display constitutive ethylene responses. In roots of *hps5*, 61 genes are upregulated relative to the wild type (WT, Columbia‐0 background). When one of the upregulated genes, that is, *AHL17* (AT5G49700) was overexpressed in WT plants under a strong constitutive *CaMV 35S* promoter (Figure S1B), the transgenic plants (*35S::AHL17*) exhibited enhanced root hair production, in terms of both root hair length and root hair density (Figure 1a–b). The increased root hair density in the overexpressing lines suggested that some of the non‐hair cells (atrichoblasts) were transformed into root hairs. This inference was confirmed by histological analyses (Figure 1d–e) and quantification of ectopically produced root hairs (Table S1). AHL28 (AT1G14490) is the closest homolog of AHL17 in the Arabidopsis AHL protein family (Figure S1A). The overexpression of *AHL28* (Figure S1B) also caused ectopic root hair formation and increased root hair length and root hair density (Figure 1a,c–e, Table S1). The length of primary roots and root epidermal cells of *35S::AHL17* and *35S::AHL28* plants did not differ from those of the WT (Figure S2), excluding the possibility that the increased root hair density in the transgenic plants was due to the change of the length of epidermal cells.

We next examined the root hair phenotypes of the single and double mutants of *AHL17* and *AHL28*, which were generated by the CRISPAR/Cas9 technique (Figure S3A–B). Root hair development of these single and double mutants did not significantly differ from that of the WT (Figure S3C), suggesting that there might be genetic redundancy among AHL17, AHL28, and other AHL proteins.

### 2.2 Gene expression patterns and subcellular localization of AHL17

**FIGURE 2:**
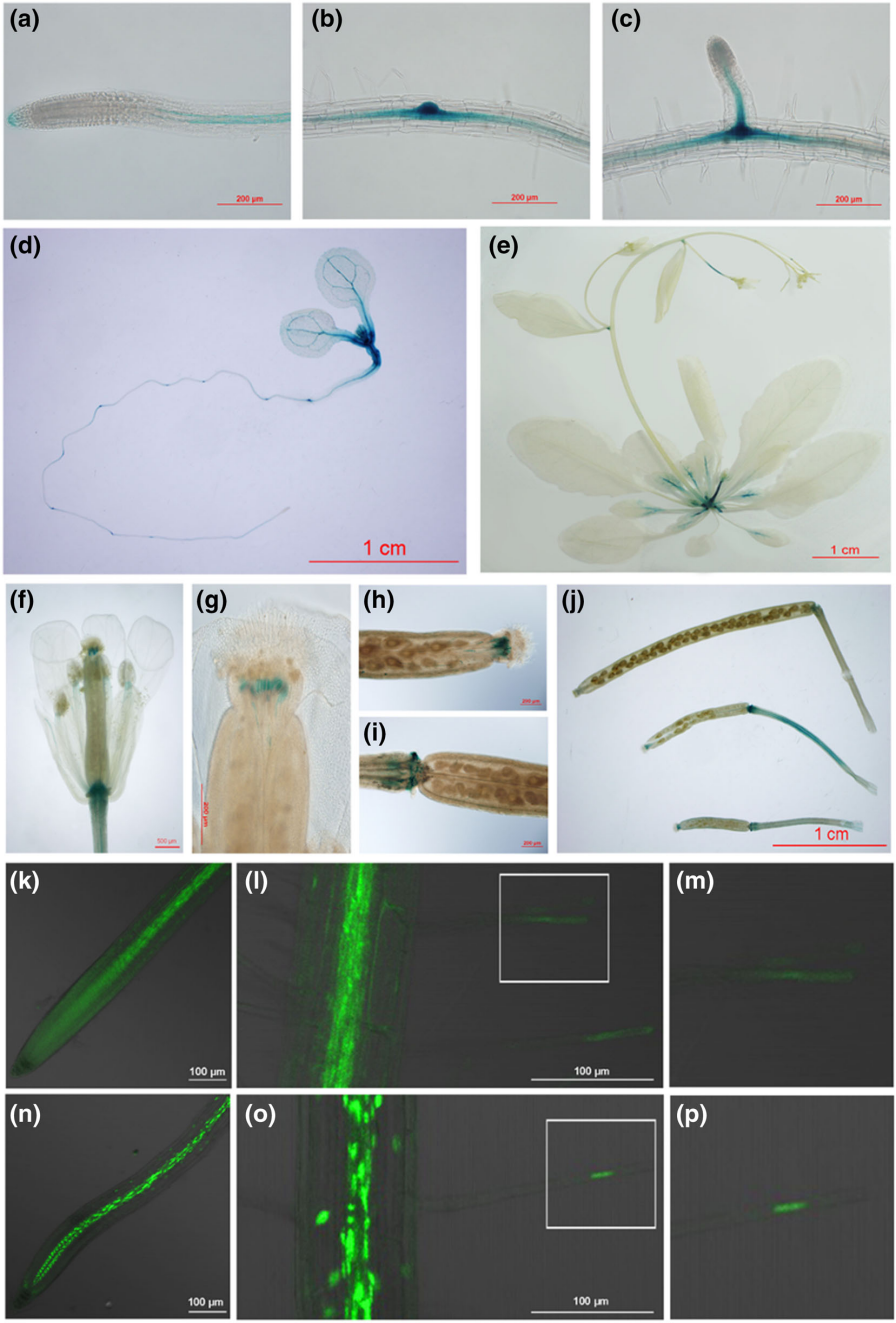
Expression patterns and subcellular localization of *AHL17*. (a–j) Histochemical staining of the GUS activity in the *AHL17::GUS* transgenic line. (a) A primary root. (b) A root segment with a lateral primordium. (c) A lateral root. (d) A 7‐day‐old seedling. (e) A 25‐day‐old plant. (f) A flower. (g) A stigma. (h) Top and (i) bottom parts of a mature silique. (j) Three siliques of different developmental stages. (k–p) Confocal microscopy images of the root of 7‐day‐old *AHL17::GFP* (k–m) and *AHL17::AHL17‐GFP* (n–p) seedlings. (m) and (p) A magnified view of the part of a root hair (enclosed in a square) of *AHL17::GFP* (l) and *AHL::AHL17‐GFP* (o) seedlings, respectively.

Before investigating the molecular functions of AHL17, we first determined the expression patterns of the *AHL17* gene and the subcellular localization of AHL17 protein. A 1.2‐kb promoter sequence of *AHL17* was fused to the GUS reporter gene and introduced into WT Arabidopsis plants. Eleven independent *AHL17::GUS* lines were generated, and the results from a representative line are presented here. *AHL17::GUS* was mainly expressed in the vascular tissues of primary and lateral roots and young leaves (Figure 2a–e). It was also expressed in the stigma and at the junctions between flower/silique and pedicles (Figure 2f–j). No GUS activity was detected in root apical meristem and root hair cells. A similar vascular tissue‐preferred expression pattern was observed in the roots of the transgenic gene when the GUS gene was replaced with a GFP gene (Figure 2k). In the *AHL17::GFP* lines, however, GFP signal could also be detected in root apical meristem and throughout a whole root hair (Figure 2l–m). This was probably because the transcription of *AHL17* was very low and the GFP protein was more stable than the GUS protein. We then generated the transgenic lines expressing the *AHL17‐GFP* fusion gene under *AHL17*'s own promoter. In the roots of these lines, the AHL17‐GFP fusion protein was localized in the nucleus of vascular and epidermal cells, including root hairs (Figure 2n–p). Use of the *CaMV 35S* promoter to overexpress the *GFP*‐*AHL17* fusion gene in WT plants (*35S::GFP‐AHL17*) also resulted in an overproduction of root hairs, indicating that the GFP‐AHL17 fusion protein was functional (Figure S4A–B). In these plants, GFP‐AHL17 was localized in the nucleus of all root cells (Figure S4C).

Because the expression of *AHL17* is upregulated in *hps5*, which displays constitutive ethylene responses in roots (Song et al., 2016), we wondered whether the expression of *AHL17* is induced by ethylene. RT‐qPCR analysis of the WT seedlings and histochemical staining of *AHL17::GUS* seedlings both showed that the expression of *AHL17* was induced by ACC, a precursor for ethylene biosynthesis (Figure S5A–B). Consistently, the *AHL17::AHL17‐GFP* lines exhibited enhanced GFP signal under ACC treatment, indicating that the accumulation of AHL17 proteins was also enhanced by ethylene (Figure S5C–D).

### 2.3 AHL17 and AHL28 enhance root hair production via RHD6

**FIGURE 3:**
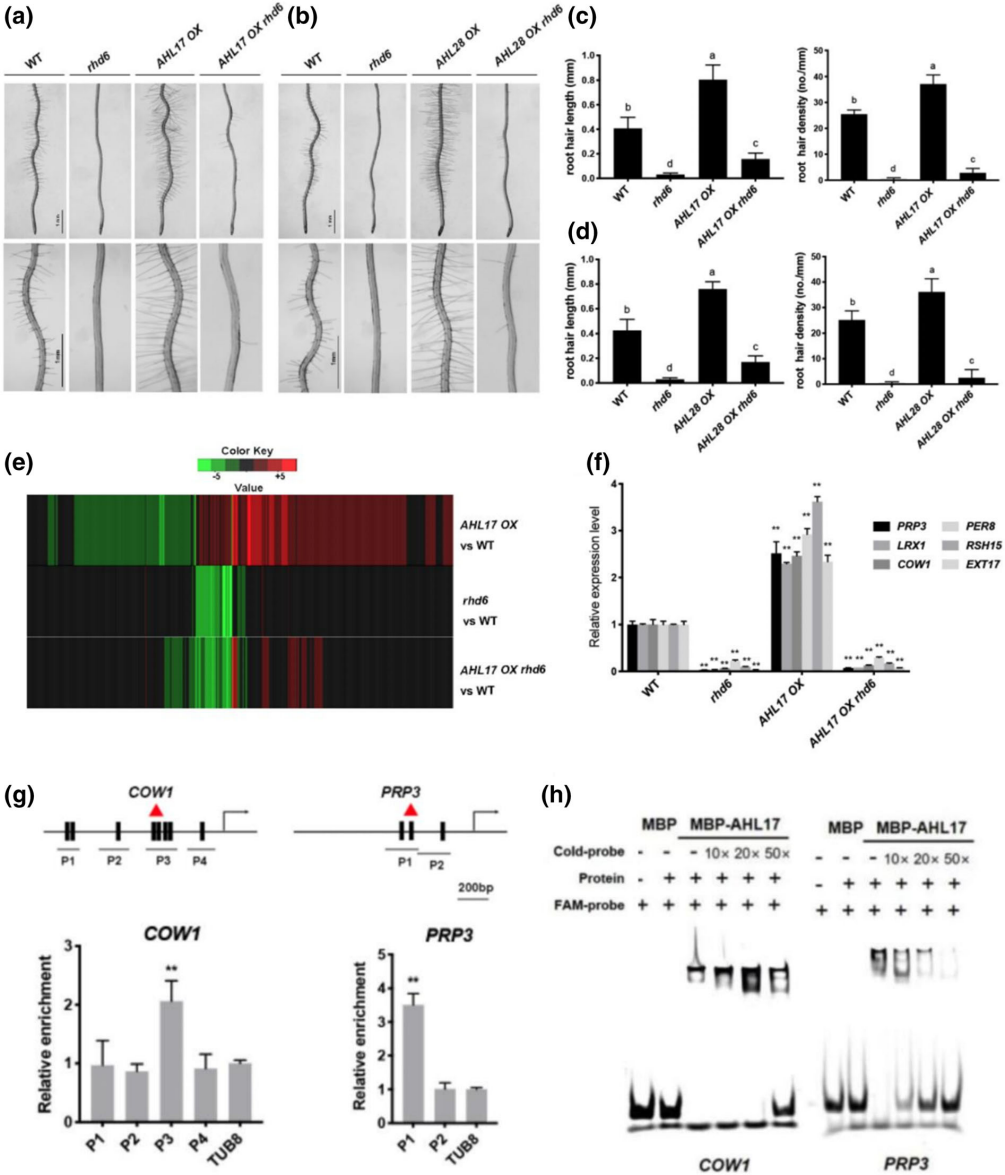
Effects of *AHL17* overexpression are suppressed by *RHD6* mutation. (a)–(d) Root hair phenotypes of 7‐day‐old seedlings of the WT, *rhd6*, *AHL17 OX*, and *AHL17 OX rhd6*, respectively. In (c) and (d), a one‐way ANOVA analysis was carried out for the whole dataset and post hoc comparisons were conducted using the SPSS Tukey HSD test at *P* < .05 level. (e) Hierarchical clusters displaying the differentially expressed genes in the 7‐day‐old seedlings of *AHL17 OX*, *rhd6*, and *AHL17 OX rhd6* lines compared with the WT. (f) Relative expression of six selected genes in the 7‐day‐old WT, *rhd6*, *AHL17 OX*, and *AHL17 OX rhd6* seedlings as determined by qPCR. Values are means ± SD of three technical replications in one experiment. The experiment was repeated three times with similar results. The expression of each of these genes in the WT was set to 1.0. Asterisks indicate a significant difference from the WT (*t*‐test, **P < .01). (g) ChIP‐qPCR assays of the binding of AHL17 to the promoters of the *COW1* and *PRP3* genes. Chromatins from *35S::GFP‐AHL17* transgenic plants were isolated and immuno‐precipitated with GFP antibodies. The levels of enrichment of the precipitated DNA fragments were quantified by qPCR assays. The ChIP signal was normalized to *TUB8* gene. Values are means ± SD of three technical replicates. Experiments were repeated two times with similar results. Asterisks indicate a significant difference from that of *TUB8* gene (*t*‐test, *P < .05, **P < .01). Schematic diagrams showing the putative AT‐hook binding elements in the promoters of these two genes are placed on the top. Black vertical lines indicate the putative AT‐hook binding elements. Letters *P* represent the PCR fragments. (h) EMSAs show the binding of AHL17 to the putative AT‐hook binding elements in the promoters of the *COW1* and *PRP3* genes. The relative positions of the probes used in EMSAs in each promoter are indicated with red triangles in (g). The unlabeled probe (cold probe) with different concentrations was used as a competitor.

RHD6 is the key regulator of the signaling cascade below GL2 and plays a critical role in root hair initiation and elongation (Franciosini et al., 2017; Huang & Zheng, 2014). Downstream of RHD6 are the proteins that directly participate in cell expansion, resulting in root hair initiation and elongation (Grierson et al., 2014; Ishida et al., 2008). To determine whether the function of AHL17 in root hair production depended on RHD6, we crossed the *35S::AHL17* plant (hereafter, referred as *AHL17 OX* plant) with *rhd6* and generated progeny that were homozygotes for both the *35S::AHL17* transgene and the *rhd6* allele (*AHL17 OX rhd6* line). These plants showed strong defects in root hair production, which were similar to the defects of *rhd6* in terms of both root hair length and root hair density (Figure 3a,c). These results demonstrated that enhanced production of root hairs caused by overexpression of *AHL17* depended on RHD6. A similar result was obtained for *AHL28* (Figure 3b,d).

### 2.4 Overexpression of *AHL17* and *AHL28* enhances the transcription of root hair‐related genes downstream of RHD6

To understand why the function of *AHL17* in root hair production was dependent on RHD6, we compared the gene transcription profiles among the WT, *35S::AHL17*, *rhd6*, and *AHL17 OX rhd6*. The cDNA libraries were constructed using the total RNA isolated from the roots of 7‐day‐old seedlings. For each genotype, three biological replicates were used. Log2 ≥ 1 or ≤−1 (2‐fold change in expression levels) and FDR (false discovery rate) ≤.01 were used as the cut‐off for the selection of the genes whose expression levels significantly differed from those of the WT. In the *AHL17 OX* line, there were 544 genes whose expression was upregulated and 361 genes whose expression was downregulated compared with the WT (Table S2). GO term analysis indicated that the differentially expressed genes are enriched in several biological processes, including trichoblast differentiation, NADPH regeneration, photosynthesis, and response to vitamin (Table S3). Among the genes related to trichoblast differentiation were those involved in the formation of Ca^2+^ gradient in the root tip, synthesis and modifications of cell wall components, small G protein‐mediated signal transduction, cytoskeleton rearrangement, and ROS production.

In *rhd6*, there were 34 genes whose expression was upregulated and 313 genes whose expression was downregulated compared with the WT (Figure 3e and Table S2). The gene expression heatmap clearly showed that almost all of the downregulated genes in *rhd6* were upregulated in the *AHL17 OX* line (Figure 3e). We then compared the RNA expression profiles among *AHL17 OX*, *rhd6*, and *AHL17 OX rhd6* lines. We found that 81 genes whose expression was upregulated in *AHL17 OX* but downregulated in *rhd6* were also downregulated in the *AHL17 OX rhd6* line (Figure 3e and Table S4). Among these 81 genes, some genes, including *COW1* (Grierson et al., 1997), *PRP3* (Bernhardt & Tierney, 2000), *LRX1* (Baumberger et al., 2003), six *RHS (ROOT HAIR‐SPECIFIC)* (Won et al., 2009), and five *EXTENSIN* (Velasquez et al., 2011), have been experimentally demonstrated to be involved in root hair development. There were also other cell wall proteins, cell wall modification enzymes, peroxidases, and so forth. Hereafter, collectively, these genes are referred as RHD6‐downstream root hair‐related genes. To confirm the results of the RNA‐seq experiment, we performed qRT‐PCR to analyze the expression of six root hair‐related genes, that is, *LRX1*, *COW1*, *PRP3*, *RSH15*, *PER8*, and *EXT17*, in different genotypes. The expression patterns of these genes shown by qRT‐PCR experiments (Figure 3f) and RNA‐seq (Table S4) were consistent, indicating that the RNA‐seq experiments were set up properly.

The above results suggested that AHL17 enhanced root hair production through increasing the transcription of root hair‐related genes downstream of RHD6.

### 2.5 AHL17 and AHL28 bind to the promoters of RHD6‐downstream genes

Next, we determined whether AHL17 and AHL28 could bind to the promoters of RHD6‐downstream genes. We first analyzed DNA sequences of the promoters of *COW1* and *PRP3* by PlantPan 3.0 (http://plantpan.itps.ncku.edu.tw/index.html). The potential AT‐hook binding motifs were identified in the promoters of these two genes (Figure 3g and Table S5). We then conducted chromatin immunoprecipitation assays followed by quantitative PCR (ChIP‐qPCR) in the roots of 7‐day‐old *35S::GFP‐AHL17* seedlings. Chromatin was isolated, cross‐linked, and precipitated with anti‐GFP antibodies. The DNA fragments that precipitated with GFP‐AHL17 proteins were analyzed by qPCR. The primers were designed to amplify the regions that contain one or more putative AT‐hook binding motifs. The results showed that some of the motifs in the promoters of these two genes amplified by qPCR were significantly enriched compared with that of the *TUB8* gene (Figure 3g), suggesting that AHL17 could bind to the promoters of RHD6‐downstream genes in vivo.

To further confirm the physical association of the AHL17 and AHL28 proteins with the promoter of *COW1* and *PRP3*, we carried out electrophoretic mobility shift assays (EMSA) using the full‐length recombinant AHL17 and AHL28 proteins produced in *E. coli* cells. The recombinant AHL17 protein contained an MBP (maltose‐binding protein) tag at its N‐terminus and a 6× His tag at its C‐terminus (MBP‐AHL17‐His), whereas the recombinant AHL28 protein only contained an MBP tag at its N‐terminus (MBP‐AHL28). The DNA probes used in EMSA were sequences that displayed high binding affinity to AHL17 in the ChIP assays (the sequences of the DNA probes are listed in Table S1). The results of EMSA indicated that the recombinant MBP‐AHL17‐His protein, as well as MBP‐AHL28 but not MBP protein alone, could bind to the FAM‐labeled DNA probes (Figures 3h and S6). The binding of recombinant AHL17 and AHL28 proteins to the FAM‐labeled probes could be reduced by unlabeled probes, indicating that the binding is sequence‐specific.

### 2.6 AHL17 and AHL28 do not affect transcription and protein accumulation of RHD6

RNA‐seq data showed that the transcription of *RHD6* and other transcription factors that regulate root hair development was not significantly altered in the *AHL17 OX* line (Table S2). We therefore wondered whether AHL17 and AHL28 affected the level of RHD6 protein. The WT, *35S::AHL17*, and *35S::AHL28* plants were crossed to the plant that carried the construct of *RHD6::RHD6‐GFP*. In the resultant F_1_ plants, the fluorescence signals of RHD6‐GFP in the plants with *35S::AHL17* or *35S::AHL28* background did not significantly differ from that in the WT background (Figure S7A). We then performed a Western blot experiment using the proteins extracted from the roots of plants carrying the *RHD6::RHD6‐GFP* construct in the WT, *35S::AHL17*, or *35S::AHL28* background using anti‐GFP antibodies. The results showed that the levels of RHD6‐GFP were not affected by the overexpression of *AHL17* or *AHL28* (Figure S7B).

### 2.7 AHL17 and AHL28 do not directly interact with RHD6

Next, we used the luciferase complementation imaging (LCI) technique (Chen et al., 2008) to determine whether AHL17 and AHL28 enhanced the expression of RHD6‐downstream genes via direct protein–protein interactions with RHD6. We first investigated whether AHL17 and AHL28 could interact with themselves and with each other. The coding sequences (CDS) of *AHL17* and *AHL28* were fused to the N‐terminal (nLUC) or C‐terminal half (cLUC) of the *LUC* gene and were co‐transformed into the leaves of *N. benthamiana* with proper controls. The co‐expression of *cLUC‐AHL17* and *AHL17‐nLUC*, *cLUC‐AHL28* and *AHL17‐nLUC*, *cLUC‐AHL17* and *AHL28‐nLUC*, or *cLUC‐AHL28* and *AHL28‐nLUC* all resulted in a strong fluorescence signal from reconstituted LUC activity (Figure S8A–B). In contrast, no reconstituted LUC signals were seen for the co‐expression of *cLUC‐AHL17* and *nLUC*, *cLUC* and *AHL17‐nLUC*, *cLUC‐AHL28* and *nLUC*, or *cLUC* and *AHL28‐nLUC*. These results indicated that AHL17 and AHL28 could interact with each other as well as with themselves. We then assessed the interaction between AHL17 and AHL28 with RHD6, RSL1, RSL2, RSL4, and GL2. The CDS of these transcription factors were fused to the N‐terminal or C‐terminal half of the *LUC* gene and co‐transformed with corresponding AHL17‐ and AHL28‐fused LUC constructs into the leaves of *N. benthamiana*. No signals of reconstituted LUC activity were detected in any of these combinations, indicating that AHL17 and AHL28 did not directly interact with RHD6 and other transcription factors (Figure S8A–B). The absence of protein–protein interactions between RHD6 with AHL17 or between AHL28 with RHD6 was confirmed by bimolecular fluorescence complementation (BiFC) assays in the leaves of *N. benthamiana* (Figure S8C).

### 2.8 AHL17 and AHL28 directly interact with HSP70 proteins

Because AHL17 did not directly interact with RHD6, we wanted to identify the missing component that linked AHL17 and RHD6. An immunoprecipitation (IP) assay was performed using total proteins extracted from 7‐day‐old seedlings of *35S::GFP‐AHL17*. The proteins in the extracts were precipitated with anti‐GFP antibodies and were subjected to mass spectrometer analysis. Among the precipitated proteins, AHL17 and AHL28 had the first and third broadest peptide spectrum matches of the peptides that resulted from trypsin digestion (Table S6). These results further suggested that AHL17 and AHL28 function as a complex. Five other AHL family proteins were also co‐precipitated with AHL17, including AHL15, 19, 22, 24, and 27 (Table S6). At least one peptide, which was specific to each AHL protein, was detected in the IP assay. All of these AHL proteins belong to the same clade in the AHL family of Arabidopsis (Figure S1A). Interestingly, the precipitated proteins with the second, fourth, and fifth highest coverage scores were HEAT SHOCK PROTEIN 70‐1 (HSP70‐1, AT5G02500), HSP70‐2 (AT5G02490), and HSP70‐4 (AT3G12580), respectively (Table S6). These proteins have also been annotated as the subunit 37e, 37d, and 37c of the plant Mediator complex (Mathur et al., 2011).

**FIGURE 4:**
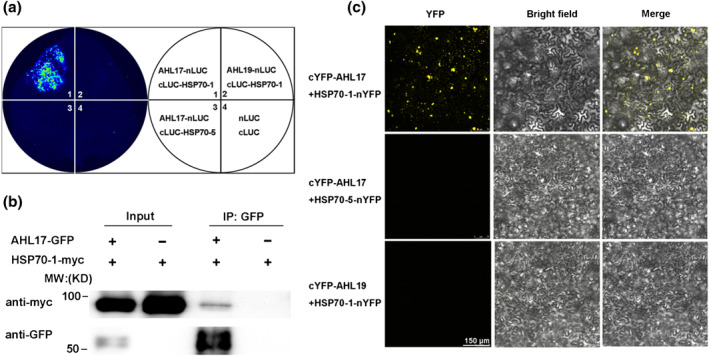
Interactions between AHL17 and HSP70‐1. (a) LCI assays. Agrobacterium carrying the construct *AHL17‐nLUC* and *cLUC‐HSP70‐1* or *cLUC‐HSP70‐5* were co‐infiltrated into the leaves of *N. benthamiana*. The constructs of *AHL19‐nLUC* and *cLUC‐HSP70‐1*, as well as the *nLUC* and *cLUC*, were also co‐infiltrated into the same leaf as the control. LUC activity was detected 2 days after infiltration. (b) Co‐IP assay. Total proteins were extracted from *N. benthamiana* leaves co‐transformed with *35S::AHL17‐GFP* and *35S::HSP70‐1‐myc* and immunoprecipitated by anti‐GFP beads. The precipitated proteins were analyzed by western blot with anti‐GFP and anti‐myc antibodies. IP: immunoprecipitated. (c) BiFC assays. The construct *cYFP‐AHL17* was co‐infiltrated with *HSP70‐1‐nYFP* or *HSP70‐5‐nYFP* into the leaves of *N. benthamiana.* The fluorescence signals of YFP were examined 2 days after infiltration. The co‐infiltration of *cYFP‐AHL19* and *HSP70‐1‐nYFP* was used as the control.

To verify the physical interactions between AHL17 and these HSP70s *in planta*, we performed LIC assays in the leaves of *N. benthamiana*. The CDS of *AHL17* was fused to the N‐terminal half of LUC (*AHL17‐nLUC*), and the CDS of *HSP70‐1* was fused to the C‐terminal half of LUC (*cLUC‐HSP70‐1*). The leaves that were co‐transformed with *AHL17‐nLUC* and *cLUC‐HSP70‐1* displayed strong fluorescence, whereas those co‐transformed with *AHL17‐nLUC* and *cLUC‐HSP70‐5* or *AHL19‐nLUC* and *cLUC‐HSP70‐1* displayed no fluorescence, demonstrating that the interaction between AHL17 and HSP70‐1 in vivo was sequence‐specific (Figure 4a). Next, we performed co‐immunoprecipitation (Co‐IP) assays in leaves of *N. benthamiana* that co‐expressed myc‐tagged HSP70‐1 (HSP70‐1‐myc) and GFP‐tagged AHL17 (AHL17‐GFP). The results showed that the HSP70‐myc protein could be co‐precipitated with AHL17‐GFP by anti‐GFP antibodies (Figure 4b). The interaction between HSP70s and AHL17 was further confirmed in the nucleus of the leaves of *N. benthamiana* by BiFC assays (Figure 4c).

The interactions of AHL17 with HPS70‐2 and HSP70‐4, but not with HSP70‐5, were also demonstrated using LIC assays (Figure S9A–B). Furthermore, we found that the interactions between AHL17 and HSP70‐1/HSP70‐2 were much stronger than the interactions between AHL17 and HSP70‐4 (Figure S9C). Similarly, AHL28 could interact with HSP70‐1, HSP70‐2, and HSP70‐4, and the interaction of AHL28 with HSP70‐1 and HSP70‐2 was stronger than with HSP70‐4 (Figure S9D–G). The interactions between AHL17 and HSP70‐2 and between AHL28 and HSP70‐1 or HSP70‐2 were further confirmed using BiFC assays (Figure S9H).

To determine which part of AHL17 interacts with HSP70, we divided AHL17 into three parts: (1) the N‐terminal half, which contained the entire AT‐hook domain and half of the PPC domain; (2) the C‐terminal half, which contained half of the PPC domain; and (3) the middle part, which contained the entire PPC domain. Using LIC assays, we found that the PPC domain but not the AT‐hook domain of AHL17 was required and sufficient for AHL17 to interact with HSP70‐1 and HSP20‐2 (Figure S10).

### 2.9 HSP70s form a complex with AHL17/28 and RHD6 *in planta*

**FIGURE 5:**
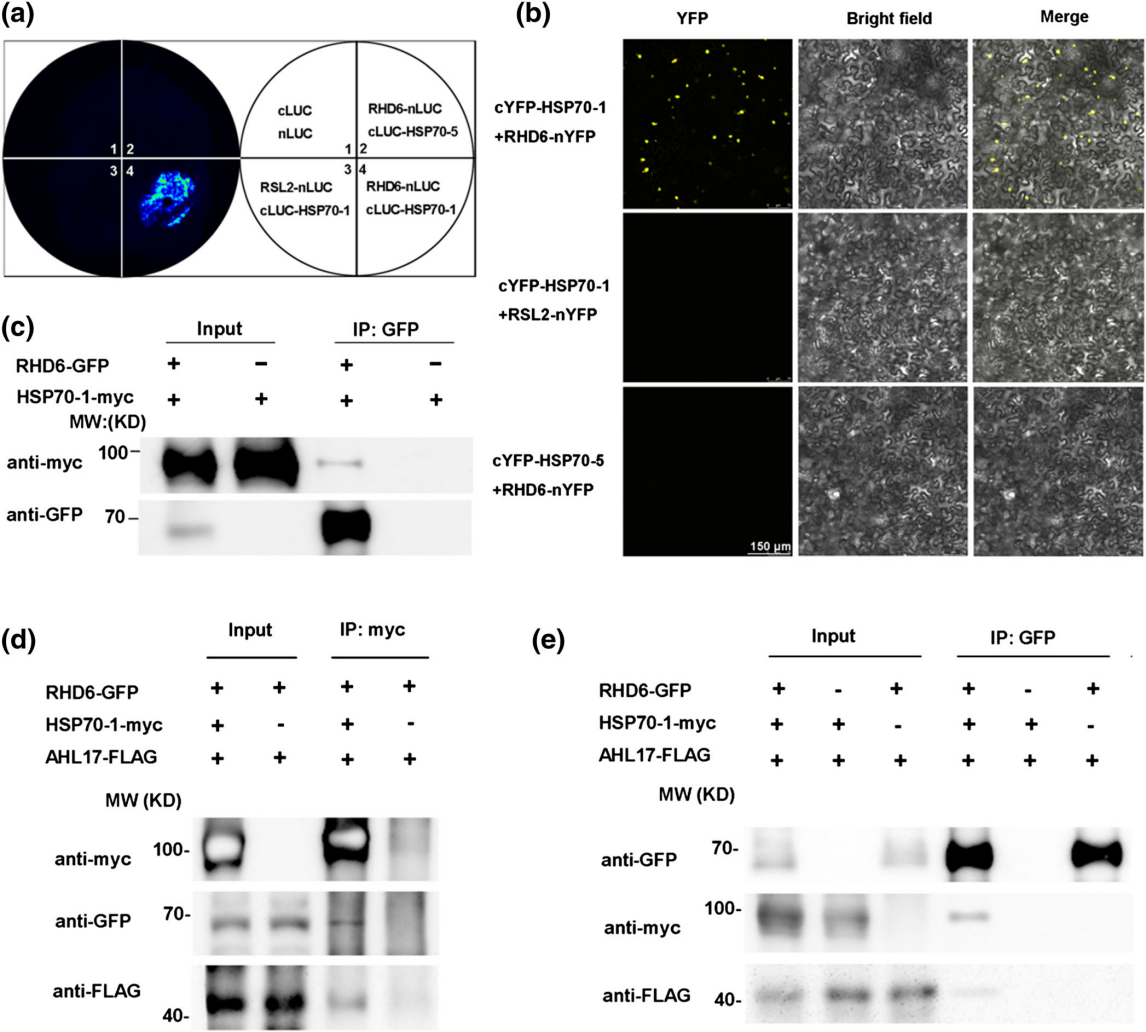
HSP70‐1 forms a complex with both AHL17 and RHD6 *in planta*. (a) LIC assays. Agrobacterium carrying the construct pairs of *cLUC‐HSP70‐1* and *RHD6‐nLUC*, *RHD6‐nLUC* and *cLUC‐HSP70‐5*, *RSL2‐nLUC* and *cLUC‐HSP70‐1*, and cLUC and nLUC were co‐infiltrated into the leaves of *N. benthamiana*. LUC activity was detected 2 days after infiltration. (b) Co‐IP assay. Total proteins were extracted from *N. benthamiana* leaves co‐transformed with the constructs of *35S::RHD6‐GFP* and *35S::HSP70‐1‐myc* and immunoprecipitated by anti‐GFP beads. The precipitated proteins were analyzed by western blot with anti‐GFP and anti‐myc antibodies. IP: immunoprecipitated. (c) BiFC assays. The construct *RHD6‐nYFP* was co‐infiltrated with *cYFP‐HSP70‐1* into the leaves of *N. benthamiana.* The fluorescence signals of YFP were examined 2 days after infiltration. The co‐infiltration of *cYFP‐HSP70‐1* and *RSL2‐nYFP* or *cYFP‐HSP70‐5* and *RHD6‐n‐YFP* was used for comparison. (d) and (e) Co‐IP assay. Total proteins were extracted from *N. benthamiana* leaves co‐transformed with the constructs of *35S::RHD6‐GFP*, *35S::HSP70‐1‐myc*, and *35S::AHL17‐FLAG* and then immunoprecipitated by anti‐myc beads (d) or anti‐GFP beads (e). The precipitated proteins were analyzed by western blot using anti‐GFP, anti‐myc, and anti‐FLAG antibodies.

Because the mutations of *RHD6* could suppress the effects of overexpression of *AHL17* on root hair production and gene transcription and because HSP70 proteins interact with AHL17, we wondered whether HSP70 proteins serve as the molecular links that enable AHL17 and RHD6 to form a complex *in planta*. To test this hypothesis, we first demonstrated the interactions between HSP70‐1 and RHD6 using LIC assays in the leaves of *N. benthamiana* (Figure 5a). The interaction was further confirmed by BiFC assay (Figure 5b) and Co‐IP assays (Figure 5c) performed in the leaves of *N. benthamiana*.

Next, we conducted Co‐IP assays to determine whether HSP70‐1 could form a complex with AHL17 and RHD6 *in planta*. We co‐expressed RHD6‐GFP, HSP70‐1‐myc, and AHL17‐FLAG in the leaves of *N. benthamiana*. First, anti‐myc beads were used to immuno‐precipitate HSP70‐1‐myc protein. Both RHD6‐GFP and AHL17‐FLAG protein could be co‐precipitated as detected by anti‐GFP and anti‐FLAG antibodies, respectively (Figure 5d). This result reconfirmed the interactions between HSP70‐1 and AHL17, as well as the interactions between HSP70‐1 and RHD6. Second, we used anti‐GFP beads to immune‐precipitate RHD6‐GFP protein. We found that the AHL17‐FLAG proteins could be co‐precipitated in the presence of HSP70‐1‐myc but that AHL17‐FLAG could not be co‐precipitated by RHD6‐GFP in the absence of HSP70‐1‐myc (Figure 5e). These results demonstrated that HSP70‐1 could form a complex with both AHL17 and RHD6 *in planta*.

## 3 DISCUSSION

Gene transcription is the first step of the Central Dogma that governs the activities of all living organisms. The regulatory mechanisms for gene transcription have been a fundamental question in biology and have been extensively studied. A general view of the protein factors required for the initiation of gene transcription includes an RNA polymerase, several general transcription factors, and certain gene‐specific transcription factors. In the past two decades, AHL proteins emerged as a new regulatory component for gene transcription. However, its exact working mechanism, especially in higher plants, is still largely unknown. In this research, we used root hair production as a readout to investigate how AHL17 and AHL28 regulate gene transcription.

Previous work has shown that AHL proteins can affect gene transcription by binding to the AT‐rich sequence in the matrix attachment regions or the promoters of their target genes (Matsushita et al., 2007; Ng et al., 2009; Xiao et al., 2009; Xu et al., 2013; Yun et al., 2012). Such binding may induce the changes of chromatin structure that allow the chromatin to adopt an open state for easy access to the general transcriptional machinery and some specific transcription factors. To facilitate gene transcription involved in hypocotyl growth, AHL27 and AHL29 have to directly interact with the transcription factors TCP4, TCP13, and TCP14 (Zhao et al., 2013). Knockout of these transcription factors suppressed the function of AHL27 and AHL29. Similarly, our research showed that AHL17 and AHL28 could bind to the AT‐rich motifs in the promoters of the RHD6‐downstream genes (Figure 3g–h) and had to act through the transcription factor RHD6 to regulate gene transcription involved in root hair development (Figure 3a–d). However, what our work differed from previous studies is that AHL17 and AHL28 do not directly interact with RHD6 (Figure S8). And, the overexpression of AHL17/AHL28 neither affects the transcription of *RHD6* nor the accumulation of *RHD6* protein (Figure S7). Therefore, there must be something missing between AHL27/AHL28 and RHD6 for their interactions to regulate gene transcription. Using the IP‐MS technique and yeast two‐hybrid experiments, we identified three HSP70 proteins as AHL17‐interacting proteins (Table S6). We further demonstrated that they could directly interact with both AHL17/AHL28 and RHD6 to form a protein complex *in planta* (Figures 4 and 5). These HSP70 proteins seem to serve as molecular glues to bring AHL17 and RHD6 together to enhance the transcription of RHD6‐downstream genes.

The HSP70 family proteins are highly conserved throughout prokaryotes and eukaryotes (Boorstein et al., 1994; Gupta & Golding, 1993). They are well known for functioning as molecular chaperons that facilitate protein folding (Boston et al., 1996; Brkljacic et al., 2009; Clément et al., 2011; Noël et al., 2007; Rosenzweig et al., 2019). The direct participation of HSP70s in the regulation of gene transcription, however, has seldom been reported in literatures. In one example with mammalian cells, HSP70 was found to regulate retinoid acid‐induced gene transcription of the retinoid acid receptor β2 by working with the Mediator complex (Gao et al., 2015). In another example, HSP70 was shown to affect the conformation of tumor suppressor p53 to regulate the transcription of the genes downstream of p53 (Dahiya et al., 2019). Before the current study, there was only one report with plants showing that HSP70 could mediate the activity of transcription factors (Tiwari et al., 2020).

Mediator is a protein complex that works with RNA polymerase II, general transcription factors (TFIIs), and certain gene‐specific transcription factors to form the pre‐initiation complex that initiates transcription (Chadick & Asturias, 2005; Dotson et al., 2000). Arabidopsis Mediator complex consists of 34 subunits, and subunits 34, 35, 36, and 37 are plant‐specific (Backstrom et al., 2007; Mathur et al., 2011; Yang et al., 2016). The different subunits of the Mediator complex are associated with specific biological processes (Yang et al., 2016). In Arabidopsis, researchers have used bioinformatics approaches to predict that HSP70‐1, ‐2, and ‐4 are the subunits MED37c, 37d, and 37e, respectively, of the Mediator complex (Mathur et al., 2011). If these three HSP70 proteins are indeed the subunits of the Mediator complex, then it is not surprising that they can participate in regulating gene transcription, although their exact working mechanism is not yet clear. We speculate that under stress conditions, HSP70s help maintain the conformation of AHL17/28 and RHD6 so that they can function properly in regulating gene transcription. To further confirm the role of HSP70s in AHL‐mediated gene transcription, it is necessary to test whether the mutation of these three *HSP70* genes can block the effects of *AHL17/28* overexpression in altering gene transcription and in promoting root hair production. These works are underway.

AHL proteins have been shown to regulate a variety of processes in plant growth and development. The functions of several AHL proteins were revealed by a gene overexpression strategy, including AHL27 and AHL29 in hypocotyl growth (Street et al., 2008), AHL27 in leaf senescence (Lim et al., 2007), and AHL22 in flowering regulation (Xiao et al., 2009). That the overexpression of *AHL17* or *AHL28* enhanced root hair production might suggest a new function of AHL proteins in plant development (Figure 1). The single and double mutants of AHL17 and AHL28, however, did not exhibit obvious defects in root hair development (Figure S3). One possibility for not seeing defects in root hair development in the *ahl17*/*ahl28* mutants is that the basal level expression of these two genes may be very low; therefore, they might not play a significant role in root hair development under normal growth conditions. However, when plants are exposed to stress conditions, such as nutrition deficiency (González‐Fontes et al., 2016; Schmidt & Schikora, 2001), mechanical stress (Okamoto et al., 2008), or bacterial infection (Galland et al., 2012), the level of ethylene is increased, which may induce the expression of *AHL17* and *AHL28*, therefore enhancing root hair production. The molecular mechanism of how ethylene regulates root hair development has also been extensively studied (Qiu et al., 2021; Song et al., 2016). In fact, we found that both transcription and protein accumulation of AHL17 are induced by ethylene (Figure S5). Like that under normal growth condition, the *ahl17*/*ahl28* mutants also did not display obvious defects in root hair production in response to ethylene treatment (Figure S3). Another explanation for this is the genetic redundancy that exists among the members of the AHL family. Genetic redundancy among the AHL proteins has been reported for AHL‐mediated hypocotyl growth (Zhao et al., 2013), flower initiation (Xiao et al., 2009), axillary meristem maturation (Karami et al., 2020), and embryo development (Karami et al., 2021). Consistent with this possibility, our IP‐MS analysis indicated that six other AHL proteins (all in the same clade of AHL protein family), including AHL28, could be co‐immunoprecipitated with AHL17 (Table S6), suggesting that some of these AHL proteins may co‐exist in the same protein complex and may perform similar functions. We used LCI assays to test whether AHL17 and AHL28 could interact with AHL19, which was co‐precipitated with AHL17 in IP assay. Indeed, the results showed that AHL17/AHL28 could interact with AHL19 (Figure S11). Currently, we are generating the knockout mutants with multiple *AHL* genes to test whether the genetic redundancy indeed exists among the members of the AHL family in regulating root hair formation.

Because of the lack of root hair phenotype of *ahl17ahl28* double mutants, we are still not certain whether the *AHL17* and *AHL28* overexpression‐enhanced root hair production is an artifact or not. However, in this work, it is more important that we revealed a previously unrecognized mechanism of AHL proteins‐mediated gene transcription rather than determining whether AHL17 and AHL28 are genuine regulators of root hair development or not. It would not be possible to reveal such a mechanism if we did not use the overexpression strategy. The overexpression strategy has been demonstrated to be very useful in revealing a new biological process or identifying new components involved in a regulatory pathway. A great example is that overexpression of a chalcone synthase gene in petunia flowers resulted in a chimera color pattern of petals, instead of a uniformly enhanced coloration of petals as usually expected (Metzlaff et al., 1997). This phenomenon led to the discovery of RNA interference in higher plants. In our previous research, we found that overexpression of a sucrose transporter gene *SUC2* in Arabidopsis using the *CaMV 35S* promoter inhibited primary root growth and enhanced plant responses to phosphate starvation, supporting that sucrose signaling is involved in these processes (Lei et al., 2011). To search for signaling components downstream of sucrose, Lei et al. (2014) screened for the genetic suppressor of *35S:SUC* plants with restored primary root growth. Unexpectedly, they found that the restored primary root growth in all suppressors was due to the silencing of the *35S* promoter activity rather than the suppression of sucrose signaling. Therefore, by serendipity, the use of these overexpressing materials resulted in the discovery of several novel components involved in gene silencing pathway (Duan et al., 2017; Lang et al., 2015; Lei et al., 2014; Nie et al., 2019; Zhou et al., 2022). Similarly, with these examples, we would like to emphasize that although the overexpression strategy used in this work was not sufficient to determine whether AHL17 and AHL28 are intrinsic regulator of root hair development, it indeed increased our understanding of the function of AHL proteins in regulating gene transcription.

Another significance of this work is that it established an ideal experimental system for further studying the functions of AHL proteins. This is because as a readout for AHL17/28's function in vivo, root hair development is very easy to score. In our future plan, we will use this system to investigate how HSP70 proteins mediate the interactions between AHL17/28 and RHD6 to regulate gene transcription. We will also test how different variants of AHL17/28 and RHD6 interact with HSP70 proteins and target DNA sequences to exert their functions, which will provide further insights into the mode of action of the AHL‐HSP70‐RHD6 complex in regulating gene transcription.

## 4 EXPERIMENTAL PROCEDURES

### 4.1 Plant materials and growth conditions

All Arabidopsis (*A. thaliana*) plants used in this study were in the Col‐0 ecotype background. The SALK T‐DNA insertional line of *rhd6* (CS877302) was obtained from the Arabidopsis Biological Resource Center (ABRC).

Arabidopsis seeds were surface sterilized in 20% (v/v) bleach for 10 min and were then washed three times with sterile‐distilled water (ddH_2_O). After being stratified at 4°C for 2 days, the seeds were sown on Petri plates containing half‐strength Murashige and Skoog (MS) medium with 1% (w/v) sucrose, .1% (w/v) MES, and 1.2% (w/v) agar (Sigma‐Aldrich). The plates with seeds were placed vertically in a growth room with a photoperiod of 16 h of light and 8 h of dark at 22–24°C. The light intensity was 100 μmol m^−2^ s^−1^. *Nicotiana benthamiana* plants were grown in soil under the same lighting conditions.

### 4.2 Root hair observation and measurement

Images of root hairs on the agar plates were captured with a stereomicroscope (Olympus SZ61) equipped with a digital camera (Olympus DP72) with 1.5× or 4.5× magnification. The lengths of root hairs located 5 to 6 mm from the root tip were measured at 1.5× magnification with the assistance of Digimizer software. For root hair numbers, both mature and immature root hairs including bulges in the region 5 to 6 mm from the root tip were counted. At least 15 seedlings for each genotype were used for root hair measurements.

To quantify root hair cells and non‐root hair cells in the root epidermis, two areas along the root of a 7‐day‐old seedling were selected randomly and were examined at 4.5× magnification. For each region, five contiguous cells from an H file and five contiguous cells from a neighboring N file were examined, and the numbers of hair cells and non‐hair cells were recorded.

### 4.3 Histological analysis of root hair formation

The histological analysis of root hair formation was performed according to Hou et al. (2021). Basically, the roots of 5‐day‐old seedlings were sectioned with a Leica microtome (Leica EM UC7). The sections were stained with periodic acid‐Schiff's reagent and observed using a differential interference contrast microscope (Nikon 80i).

### 4.4 Vector construction and plant transformation

To generate plant overexpressing vectors of *35S::AHL17* and *35S::AHL28*, the genomic sequences of the corresponding genes were amplified from genomic DNA of WT Arabidopsis plants. The genomic sequence of each gene was cloned into the *Bam*HI and *Sac*I restriction sites between the *CaMV 35S* promoter and the *NOS* terminator of the plant expression vector pZH01.

The mutated alleles of *AHL17* and *AHL28* were also generated in the WT using a CRISPR/Cas9‐based genome editing system developed by Xing et al. (2014). The targeted editing sites in the corresponding genes were determined using the online service http://www.genome.arizona.edu/crispr/CRISPRsearch.html. The two target sequences for one corresponding gene were synthesized in the DT1‐F0 to DT2‐R0 primer pair, and the intermediate pCBC‐DT1T2 fragments were amplified from the pCBC template. The DNA fragments were then cloned into the plant vector pHSE401 by the Golden Gate cloning method via the *Bsa*I restriction site. The CRISPR *ahl17* and *ahl28* constructs were transformed into WT Arabidopsis plants. The *ahl17ahl28* double mutant was generated through a genetic cross. The sequences of the primers used for PCR amplification of a DNA fragment and vector construction are listed in Table S7.

For analysis of the gene expression patterns of *AHL17*, a 1.2‐kb DNA sequence upstream of the start codon of *AHL17* was amplified by PCR from the genomic DNA of WT Arabidopsis plants. The DNA fragment was cloned into the *Xba*I and *Xma*l restriction sites in front of the GUS reporter gene on the vector pBI101. To generate the construct of *AHL17::GFP*, the *CaMV 35S* promoter sequence on the vector pJG186 was excised by *EcoR*I and *Sac*I digestion and replaced with the *AHL17* promoter. For analysis of the subcellular localization of AHL17 protein, the CDS of *AHL17* was isolated by PCR from plant cDNAs and was cloned into the sites of *Kpn* I and *Pst* I on vector pJG053 and into the sites of *Sac*I and *Apa*I on vector pJG186, resulting in the constructs *35S::GFP‐AHL17* and *35S::AHL17‐GFP*, respectively. To generate the *AHL17::AHL17‐GFP* construct, the genomic DNA sequence of *AHL17* was inserted into the vector AHL17::GFP using *Sac*I and *Apa*I restriction enzymes.

To generate vectors *RHD6::RHD6‐GFP*, the CDS of *RHD6* was first inserted into pJG186 using *Sac*I and *Apa*I restriction enzymes, which resulted in the *35S::RHD6‐GFP* construct. The *CaMV 35S* promoter on this construct was then replaced by the promoter regions of *RHD6*. All of the primers used for the construction of the vectors are listed in Table S7.

All constructs were mobilized into the *Agrobacterium tumefaciens* strain GV3101 using the freeze–thaw method and were transformed into *Arabidopsis* plants via the *Agrobacterium*‐mediated flower dip method (Clough & Bent, 1998). The stable transgenic lines were selected using antibiotic‐containing media.

### 4.5 Quantitative real‐time PCR analysis

qPCR analyses for gene expression were performed as described by Song et al. (2016). The primers used for qPCR analyses are listed in Table S7.

### 4.6 RNA‐Seq analyses

Total RNAs were extracted from the roots of 7‐day‐old seedlings using the RNeasy Plant Mini kit (Qiagen). The RNA‐seq analyses were performed at Bionova Company. The RNA‐seq libraries were constructed through adaptor ligation and were subjected to single‐ended sequencing with a 50‐nucleotide reading length. FastQC software was used to assess the quality of raw sequencing reads. The adaptor and the low‐quality reads were removed before data analysis. The remaining reads were aligned to the Arabidopsis TAIR 10.0 reference genome using TopHat2. After the sequences of rRNA or tRNA were removed, the TopHat read alignments were assembled by Cufflinks software to produce a transcriptome annotation of the genome. The expression levels for all transcripts were normalized to per million mapped reads. The differentially expressed genes were identified using DESeq2 (Love et al., 2014). The cutoff value for differentially expressed transcripts was a ≥2‐fold change in expression with an FDR ≤ 0.05.

### 4.7 Analysis of GUS activity

The histochemical analyses of GUS activity were performed as described by Jefferson (1989).

### 4.8 Confocal microscopy

The fluorescence signals produced from the roots of 7‐day‐old seedlings of the transgenic plants carrying a GFP gene or from BiFC assays (signals from YFP) were observed with a confocal microscope (Zeiss LSM710META or Leica STED). The wavelengths of excitation/emission were 488 nm/491–535 nm for GFP and 514 nm/520–550 nm for YFP proteins. The captured fluorescence images were processed and analyzed with Zen Black, Zen Blue software, or LAS X.

### 4.9 EMSAs

The full‐length CDSs of AHL28 were cloned into vector pMAL‐c5x (NEB) using *Not*I and *Sal*I restriction sites at the N terminus of an MBP tag to generate MBP‐AHL28 recombinant protein. For better purification, the full‐length CDSs of AHL17 were cloned into vector pMAL‐c5x (NEB) with a 6x His tag sequence in its C terminus, thus generating MBP‐AHL17‐His. These constructs were separately transformed into *E. coli* strain Rosseta. The MBP‐AHL28 was purified using Amylose Resin gravity flow columns (NEB), whereas the MBP‐AHL17‐His was purified using Ni‐NTA agarose columns (QIAGEN).

The FAM‐labeled hot probes containing the putative AT‐hook binding motifs and its cold probes were generated by annealing the FAM‐labeled complementary oligonucleotides. The sequences of the oligonucleotides used for generating various probes are listed in Table S7. EMSAs were performed as described in Sun et al. (2016).

### 4.10 ChIP‐qPCR assay

ChIP‐qPCR assays were performed essentially as described by Saleh et al. (2008). Briefly, the chromatins were isolated from the 14‐day‐old transgenic plants overexpressing *GFP‐AHL17*. The isolated chromatins were cross‐linked with 1% formaldehyde, sonicated, and precipitated by Anti‐GFP Magarose Beads (Smart Lifesciences). The precipitated DNA fragments were released by 200‐mM NaCl and subjected to qPCR analysis.

### 4.11 LCI and BiFC assays

For LCI assays, the full‐length CDSs of *AHL17*, *AHL28*, *AHL19*, *RHD6*, *HSP70‐1*, *HSP70‐2*, *HSP70‐4*, and *HSP70‐5* genes were amplified by PCR from plant cDNAs and were individually inserted into the vectors of pCAMBIA‐nLUC and pCAMBIA‐cLUC (Chen et al., 2008) to generate X‐nLUC and cLUC‐Y (X and Y represent any CDS) constructs. For BiFC assays, the full‐length CDSs of *AHL17*, *AHL28*, *AHL19*, *RHD6*, *RSL1*, *HSP70‐1*, *HSP70‐2*, and *HSP70‐5* genes were individually inserted into the vector of nYFP or cYFP by the Gateway cloning method. The LCI and BiFC assays were performed in the leaves of *N. benthamiana* according to Sun et al. (2016).

### 4.12 Protein extraction and western blots

The root of 7‐day‐old Arabidopsis seedlings or the leaves of *N. benthamiana* transformed with various gene constructs were ground to fine powders in liquid nitrogen and suspended in ice‐cold protein extraction buffer (50‐mM Tris·HCl [pH 7.5], 150‐mM NaCl, .1% Triton X‐100, .2% Nonidet P‐40, .6‐mM PMSF, 20‐mΜ MG132, with Roche protease inhibitor mixture). Western blots were performed according to Song et al. (2016).

### 4.13 Co‐immunoprecipitation assays

For testing the interaction between AHL17 and HSP70‐1/2 as well as the interaction between RHD6 and HSP70‐1/2, the full‐length CDSs of *HSP70‐1* and *HSP70‐2* were cloned into the vector pMYC2 (modified form pROK2) through *Xba*l and *Bam*HI restriction sites to generate *35S::HSP70‐1‐myc* and *35S::HSP70‐2‐myc* constructs. *Agrobacterium* strains carrying *35S::AHL17‐GFP* and *35S::HSP70‐1‐myc* or *35S::HSP70‐2‐myc*, as well as *35S::RHD6‐GFP* and *35S::HSP70‐1‐myc* or *35S::HSP70‐2‐myc*, were co‐infiltrated into the leaves of *N. benthamiana*. The infiltrated leaves with empty GFP construct were used as a negative control. Two days after infiltration, the infiltrated leaves were harvested, ground in liquid nitrogen, and suspended in an ice‐cold protein extraction buffer to extract the total proteins. Anti‐GFP Magarose Beads (Smart Lifesciences) were then added to the extracts, which were incubated at 4°C for 1 h with gentle shaking. The precipitated proteins were washed at least six times using ice‐cold protein extraction buffer, and bound proteins were eluted by heating the beads in SDS protein loading buffer at 95°C for 10 min. The precipitated AHL17 and RHD6 proteins were detected by Western blotting using anti‐GFP antibody (Abmart), whereas the HSP70‐1 and HSP70‐2 proteins were detected by Western blotting using anti‐myc antibody (Abmart).

Co‐IP assays for testing the association of AHL17, HSP70‐1, and RHD6 were similar except that the protein extracts contained three co‐transformation proteins, including *35S::RHD6‐GFP*, *35S::HSP70‐1‐myc*, and *35S::AHL17‐FLAG* (the CDS of *AHL17* was amplified from cDNA and inserted into modified PS1300).

### 4.14 IP‐MS experiments

For IP‐MS experiments, proteins were extracted from roots of 7‐day‐old seedlings of *35S::GFP‐AHL17* plants and were precipitated using anti‐GFP antibody (Abmart) before analysis as mentioned earlier. The IP‐MS analysis of the precipitated proteins was performed at the Center of Biomedical Analysis of Tsinghua University. WT plants were used as a negative control.

## AUTHOR CONTRIBUTIONS

Qike Zeng, Li Song, and Dong Liu designed the research. Qike Zeng, Li Song, Mingzhe Xia, Zai Zheng, Ziang Chen, and Ximing Che performed the experiments. Qike Zeng, Li Song, Mingzhe Xia, Ziang Chen, and Dong Liu analyzed the data. Qike Zeng and Dong Liu wrote the manuscript.

## CONFLICT OF INTEREST STATEMENT

The authors declare no conflict of interest.

## Supporting information


**Fig. S1.** Overexpression of *AHL17* and *AHL28* in transgenic plants.
**(A)** Phylogenetic tree of the Arabidopsis AHL family.
**(B)** Relative expression of *AHL17* and *AHL28* in the root of 7‐day‐old WT, *35S::AHL17* and *35S::AHL28* seedlings as determined by qPCR. Values are means ± SD of three technical replications in one experiment. The experiment was repeated three times with similar results. Asterisks indicate a significant difference from the WT (*t*‐test, **: P < .01).
**Figure S2.** Comparison of the seedling morphologies and root epidermal cells between the WT and *AHL17*‐ and *AHL28*‐overexpressing lines.
(A) Morphologies of 7‐d‐old seedlings of the WT, *35S::AHL17*, and *35S::AHL28* lines. (B) Length of root epidermal cells of 7‐d‐old seedlings of the WT, *35S::AHL17*, and *35S::AHL28* lines. Values are means ± SD of 15 root epidermal cells for each line. Student *t*‐test was used to analyze the difference between the WT and each overexpressing line and no significance was found (P < .01).
**Figure S3.** Root hair phenotypes of *AHL17* and *AHL28* mutants.
(A) and (B) The altered *AHL17* and *AHL28* gene sequences generated by CRISPR/Cas9 editing technology and resultant changes in their encoded proteins. Red arrows indicate the editing sites where the sequences between them are deleted. The red box indicates a premature stop codon introduced into the *AHL28* gene.
(C) Root hair phenotypes of 7‐d‐old seedlings of the WT, *ahl17*, *ahl28*, and *ahl17ahl28* grown under normal growth condition (1/2 MS), phosphate deficiency (‐Pi) and presence of .1 μM ACC and .5 μM ACC.
**Figure S4.** Root hair phenotypes of *35S::GFP‐AHL17* transgenic lines. (A) Relative expression of the *AHL17* gene in the roots of 7‐d‐old seedlings of the WT and three independent *35S::GFP‐AHL17* lines*.* Values are means ± SD of three technical replications in one experiment. The experiment was repeated three times with similar results. Asterisks indicate a significant difference from the WT (*t*‐test, **P < .01). (B) Root hair phenotypes of 7‐d‐old seedlings of the WT and three independent *35S::GFP‐AHL17* lines. (C) Confocal microscopy images of the roots of 7‐d‐old *35S::GFP‐AHL17* seedlings. The GFP signals indicated that the GFP‐AHL17 fusion proteins are localized in the nucleus.
**Figure S5.** Effects of ACC treatment on gene transcription and protein accumulation of AHL17.
(A) Relative expression of *AHL17* in 7‐day‐old seedlings of the WT grown on medium in absence (−) or presence (+) of 5 μM ACC as determined by RT‐qPCR. Values are means ± SD of three biological replications. The expression of *AHL17* in the seedlings grown on medium without ACC was set to 1.0. Asterisks indicate a significant difference from the WT (*t*‐test, **P < .01). (B) Histochemical staining of 7‐day‐old *AHL17::GUS* seedlings grown in the absence or presence of ACC. (C) Quantification of GFP fluorescence signals in seedlings shown in (D). Values are means ± SD of 15 roots of ACC‐treated or non‐treated seedlings, respectively. Asterisks indicate a significant difference from the WT (*t*‐test, **P < .01). (D) Confocal microscopy images showing the accumulation of GFP‐AHL17 proteins in 7‐d‐old seedlings in absence or presence of 5 μM ACC.
**Figure S6.** AHL28 binds to the promoters of the *COW1* and PRP3 genes.
EMSAs showing the binding of AHL28 to the putative AT‐hook binding elements in the promoters of the *COW1* and *PRP3* genes. The experiment was performed using 1.5 μg MBP‐AHL28 and 3 μg MBP proteins. The working concentration FAM‐labeled probe was 1 μM. Different amounts of excess unlabeled probe (cold probe) were added as competitors.
**Figure S7.** Accumulation of RHD6‐GFP fusion proteins in the WT, *35S::AHL17,* and *35S::AHL28* transgenic plants.
(A) Confocal microscopy images of the root tips of 7‐day‐old seedlings of F_1_ progeny derived from the cross between *RHD6::RHD6‐GFP* and WT, *35S::AHL17*, or *35S::AHL28* lines. (B) Western blot analysis of RHD6 protein accumulation in the roots of F_1_ plants derived from cross between *RHD6::RHD6‐GFP* and WT, *35S::AHL17*, or *35S::AHL28* lines. The total proteins were extracted from roots of 7‐d‐old seedlings and RHD6‐GFP were detected by Western blot analysis with anti‐GFP antibody. Actin was used as a loading control.
**Figure S8.** Interaction between AHL17/AHL28 and major root transcription factors involved in root hair development.
(A) and (B) LIC assays. A pair of constructs with various combinations (indicated in the diagrams on right) were co‐transformed into the leaves of *N. benthamiana.* LUC activity was detected 2 d after infiltration to determine the interaction between various transcription factors with AHL17 (A) or AHL28 (B). The co‐transformation of *AHL17‐nLUC* and *cLUC‐AHL28*, *cLUC‐AHL17* and *AHL28‐nLUC*, *AHL17‐nLUC* and *cLUC‐AHL17*, or *AHL28‐nLUC* and *cLUC‐AHL28* was used as a positive control. (C) BiFC assays. A pair of constructs of various combinations were co‐transformed into the leaves of *N. benthamiana.* Yellow fluorescence signals were detected 2 d after infiltration to determine the interaction between AHL17 and AHL28 with RHD6 and RSL1, respectively. The co‐transformation of *cYFP‐AHL17* and *AHL28‐nYFP* or *cYFP‐AHL28* and *AHL17‐nYFP* was used as the positive control.
Figure S9. Interaction between AHL17/AHL28 and three HSP70 proteins.
(A) to (C) LIC assays. A pair of constructs with various combinations (indicated in the diagrams on right) were co‐infiltrated into the leaves of *Nicotiana benthamiana* to examine the interactions between AHL17 with three HSP70 proteins. Luciferase activity was detected 2 d after infiltration. (A) and (B) Interactions between AHL17 and HSP70–2 and HSP70–4. (C) Relative strength of the interactions between AHL17 with HSP70–1, HSP70–2, HSP70–4, and HSP70–5. (D) to (G) LCI assays. The interactions between AHL28 and HSP70–2 (D), AHL28 and HSP70–4 (E). (F) Comparison of the relative strength of the interactions between AHL28 with HSP70–1, HSP70–2, HSP70–4, and HSP70–5. (G) Specific interactions between AHL28 and HSP70–1. (H) Specific interactions between AHL17 and HSP70–2, between AHL28 and HSP70–1, and between AHL28 and HSP70–2 as indicated by BiFC assays in *N. benthamiana*.
**Figure S10.** The protein domain of AHL17 involved in the interactions between AHL17 and HSP70–1 and HSP70–2.
(A) Diagrams showing different parts of AHL17 that were used in BiFC assays. (B) BiFC assays. A pair of constructs of various combinations (as indicated on the left and top of the panel) were co‐transformed into the leaves of *N. benthamiana.* Yellow fluorescence signals were detected 2 d after infiltration to determine the interaction between different parts of AHL17 and HSP70–1 and HSP70–2, respectively. The co‐transformation of LUC‐nYFP with cYFP‐AHL17 was used as the negative control.
**Figure S11.** Interactions between AHL17/AHL28 and AHL19.
LCI assays were used to test the interaction between AHL17/AHL28 and AHL19. The CDS of AHL17 and AHL28 were fused to the N‐terminal half (nLUC) of the LUC gene and the CDS of AHL19 was fused with the C‐terminal half (cLUC) of the LUC gene. A pair of constructs with various combinations (indicated in the diagrams on right) were co‐infiltrated into the leaves of *Nicotiana benthamiana* to examine the interactions between AHL17/AHL28 and AHL19. Luciferase activity was detected 2 d after infiltration. The co‐infiltration of the constructs of AHL17‐nLUC and AHL28‐cLUC was used as a positive control and co‐infiltration of the constructs nLUC and cLUC was used as a negative control.

**Table S1.** Specification of cell types in root epidermis of various genotypes.

**Table S2.** RNA‐seq results of AHL17 OX, rhd6, and AHL17 OX rhd6 vs the WT (Col‐0), respectively.

**Table S3.** Go term analyses of the differentially expressed genes in AHL17 OX (35S::AHL17–3), rhd6, and AHL17 OX rhd6 lines vs the WT.

**Table S4.** The genes whose expression is up‐regulated in AHL17 OX but down‐regulated in rhd6 and AHL17 OX rhd6.

**Table S5.** Putative AT‐hook binding motifs in the promotes of the COW1 and PRP3 genes analyzed by ChIP‐qPCR.

**Table S6.** Mass spectroscopy analysis of immunoprecipitated proteins.

**Table S7.** Sequences of the primers used in different experiments.

## Data Availability

The raw experimental data and the plant lines described are available upon request. Accession Numbers The gene sequences used this study can be found in The Arabidopsis Information Resource under the following accession numbers: AHL17 (AT5G49700), AHL28 (AT1G14490), AHL19 (AT3G04570), RHD6 (AT1G66470), PRP3 (AT3G62680), LRX1 (AT1G12040), COW1 (AT4G34580), RHS15 (AT4G25220), HSP70‐1 (AT5G02500), HSP70‐2 (AT5G02490), HSP70‐4 (AT3G12580), HSP70‐5 (AT1G16030), and UBQ5 (AT3G62250).
