# Supplementary material for: Overexpression of AHL proteins enhances root hair production by altering the transcription of RHD6‐downstream genes
Source: Plant Direct. 2023 Aug 2;7(8):e517. doi: 10.1002/pld3.517 (PMC10416611; doi:10.1002/pld3.517)
Supplement: Supplementary file 1 — Fig. S1. Overexpression of AHL17 and AHL28 in transgenic plants. (A) Phylogenetic tree of the Arabidopsis AHL family. (B) Relative expression of AHL17 and AHL28 in the root of 7‐day‐old WT, 35S::AHL17 and 35S::AHL28 seedlings as determined by qPCR. Values are means ± SD of three technical replications in one experiment. The experiment was repeated three times with similar results. Asterisks indicate a significant difference from the WT (t‐test, **: P < .01). Figure S2. Comparison of the seedling morphologies and root epidermal cells between the WT and AHL17‐ and AHL28‐overexpressing lines. (A) Morphologies of 7‐d‐old seedlings of the WT, 35S::AHL17, and 35S::AHL28 lines. (B) Length of root epidermal cells of 7‐d‐old seedlings of the WT, 35S::AHL17, and 35S::AHL28 lines. Values are means ± SD of 15 root epidermal cells for each line. Student t‐test was used to analyze the difference between the WT and each overexpressing line and no significance was found (P < .01). Figure S3. Root hair phenotypes of AHL17 and AHL28 mutants. (A) and (B) The altered AHL17 and AHL28 gene sequences generated by CRISPR/Cas9 editing technology and resultant changes in their encoded proteins. Red arrows indicate the editing sites where the sequences between them are deleted. The red box indicates a premature stop codon introduced into the AHL28 gene. (C) Root hair phenotypes of 7‐d‐old seedlings of the WT, ahl17, ahl28, and ahl17ahl28 grown under normal growth condition (1/2 MS), phosphate deficiency (‐Pi) and presence of .1 μM ACC and .5 μM ACC. Figure S4. Root hair phenotypes of 35S::GFP‐AHL17 transgenic lines. (A) Relative expression of the AHL17 gene in the roots of 7‐d‐old seedlings of the WT and three independent 35S::GFP‐AHL17 lines. Values are means ± SD of three technical replications in one experiment. The experiment was repeated three times with similar results. Asterisks indicate a significant difference from the WT (t‐test, **P < .01). (B) Root hair phenotypes of 7‐d‐old seedl [file PLD3-7-e517-s005.docx]

**Supplemental Figure S1**

**C**

#
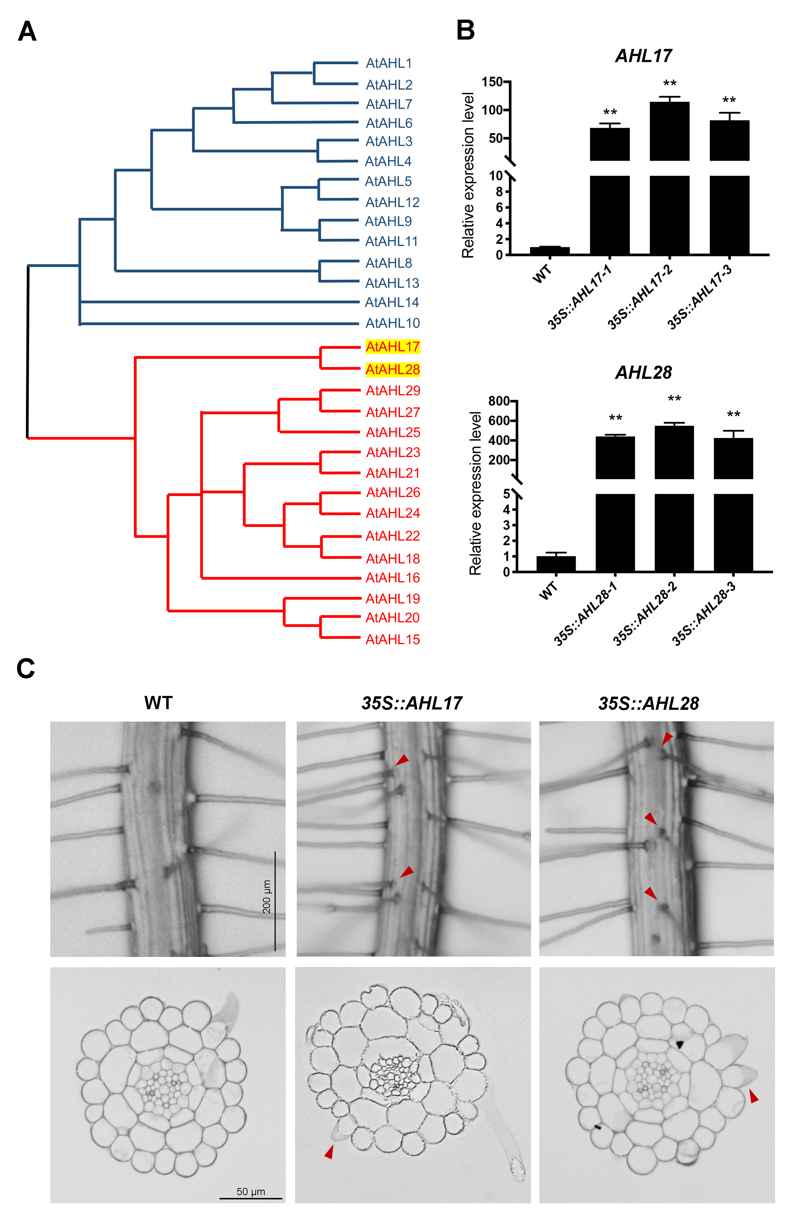


# Fig. S1 Overexpression of *AHL17* and *AHL28* in transgenic plants.

**(A)** Phylogenetic tree of the Arabidopsis AHL family.

**(B)** Relative expression of *AHL17* and *AHL28* in the root of 7-day-old WT, *35S::AHL17* and *35S::AHL28* seedlings as determined by qPCR. Values are means ± SD of three technical replications in one experiment. The experiment was repeated three times with similar results. Asterisks indicate a significant difference from the WT (*t*-test, **: P < 0.01).

**Supplemental Figure S2**

**
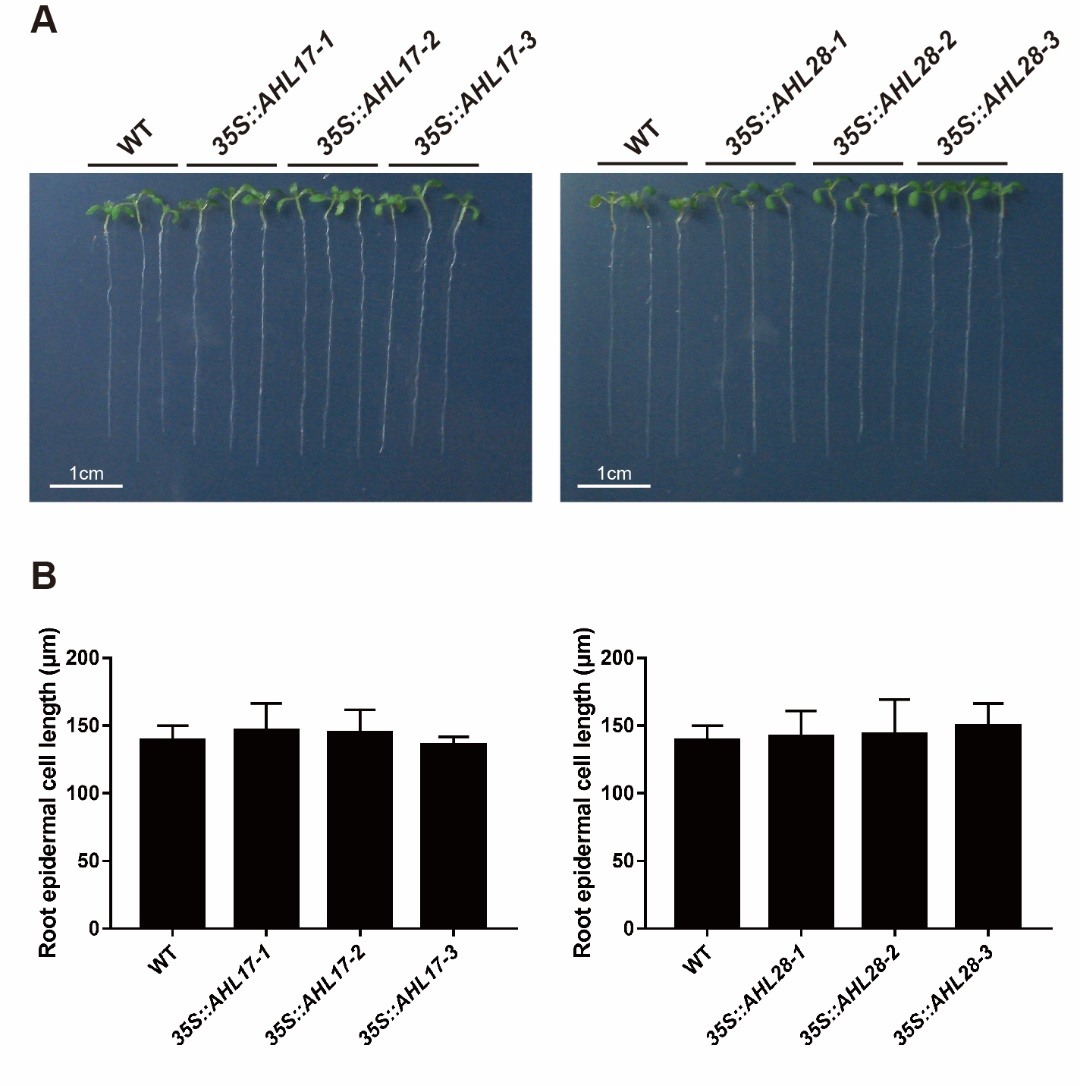
**

**Fig. S2 Comparison of the seedling morphologies and root epidermal cells between the WT and *AHL17*- and *AHL28*-overexpressing lines.**

1. Morphologies of 7-d-old seedlings of the WT, *35S::AHL17*, and *35S::AHL28* lines. (B) Length of root epidermal cells of 7-d-old seedlings of the WT, *35S::AHL17*, and *35S::AHL28* lines. Values are means ± SD of 15 root epidermal cells for each line. Student *t*-test was used to analyze the difference between the WT and each overexpressing line and no significance was found (P < 0.01).

**Supplemental Figure S3**


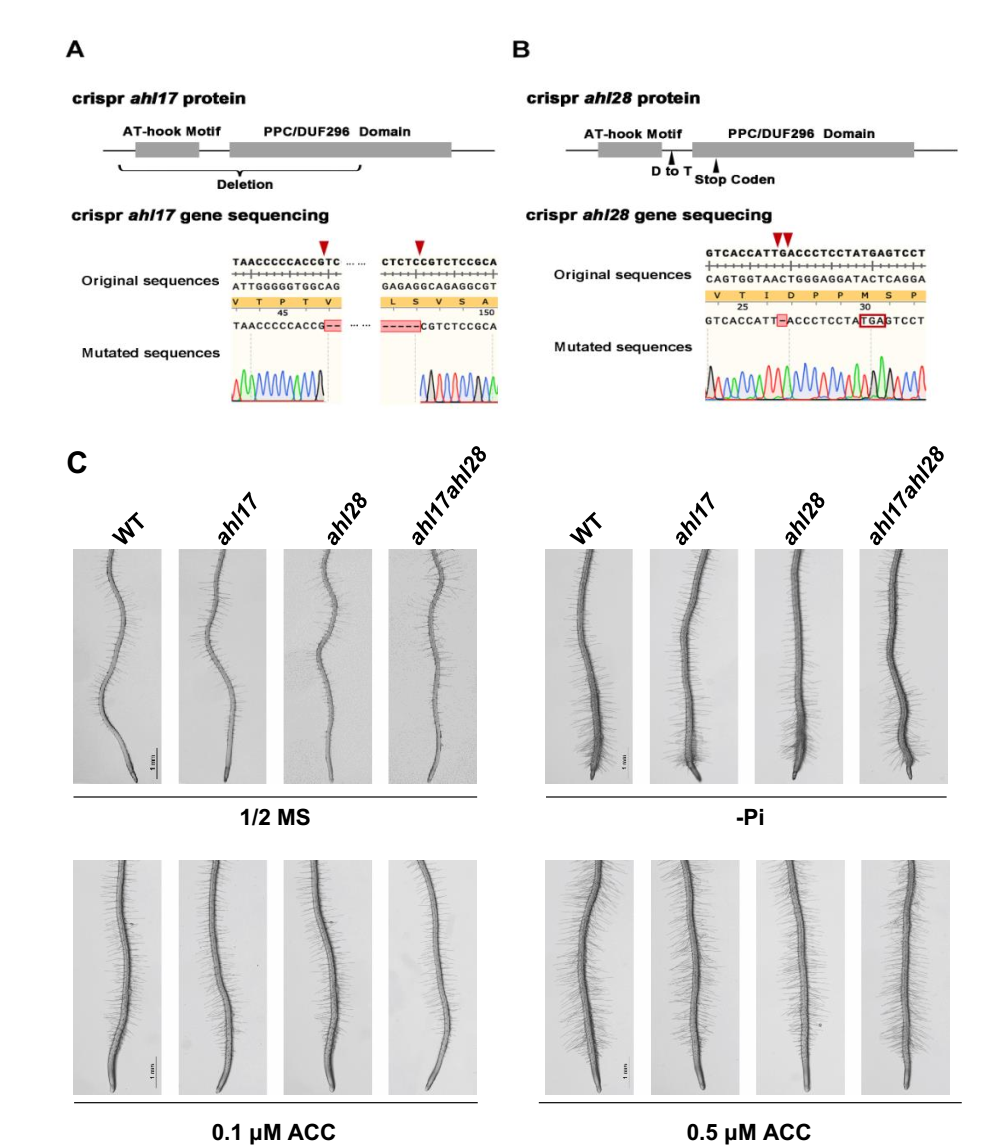


# Fig. S3 Root hair phenotypes of *AHL17* and *AHL28* mutants.

(A) and (B) The altered *AHL17* and *AHL28* gene sequences generated by CRISPR/Cas9 editing technology and resultant changes in their encoded proteins. Red arrows indicate the editing sites where the sequences between them are deleted. The red box indicates a premature stop codon introduced into the *AHL28* gene.

(C) Root hair phenotypes of 7-d-old seedlings of the WT, *ahl17*, *ahl28*, and *ahl17ahl28* grown under normal growth condition (1/2 MS), phosphate deficiency (-Pi) and presence of 0.1 μM ACC and 0.5 μM ACC.

**Supplemental Figure S4**


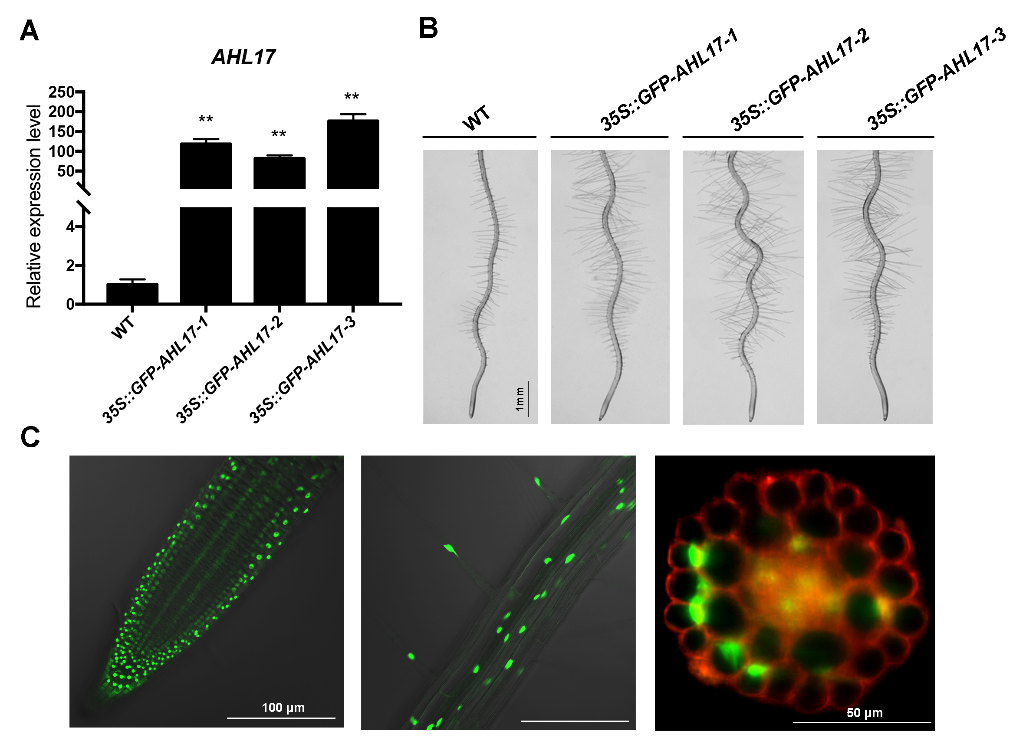


# Fig. S4 Root hair phenotypes of *35S::GFP-AHL17* transgenic lines. (A) Relative expression of the *AHL17* gene in the roots of 7-d-old seedlings of the WT and three independent *35S::GFP-AHL17* lines*.* Values are means ± SD of three technical replications in one experiment. The experiment was repeated three times with similar results. Asterisks indicate a significant difference from the WT (*t*-test, **P < 0.01). (B) Root hair phenotypes of 7-d-old seedlings of the WT and three independent *35S::GFP-AHL17* lines. (C) Confocal microscopy images of the roots of 7-d-old *35S::GFP-AHL17* seedlings. The GFP signals indicated that the GFP-AHL17 fusion proteins are localized in the nucleus.

**Supplemental Figure S5**


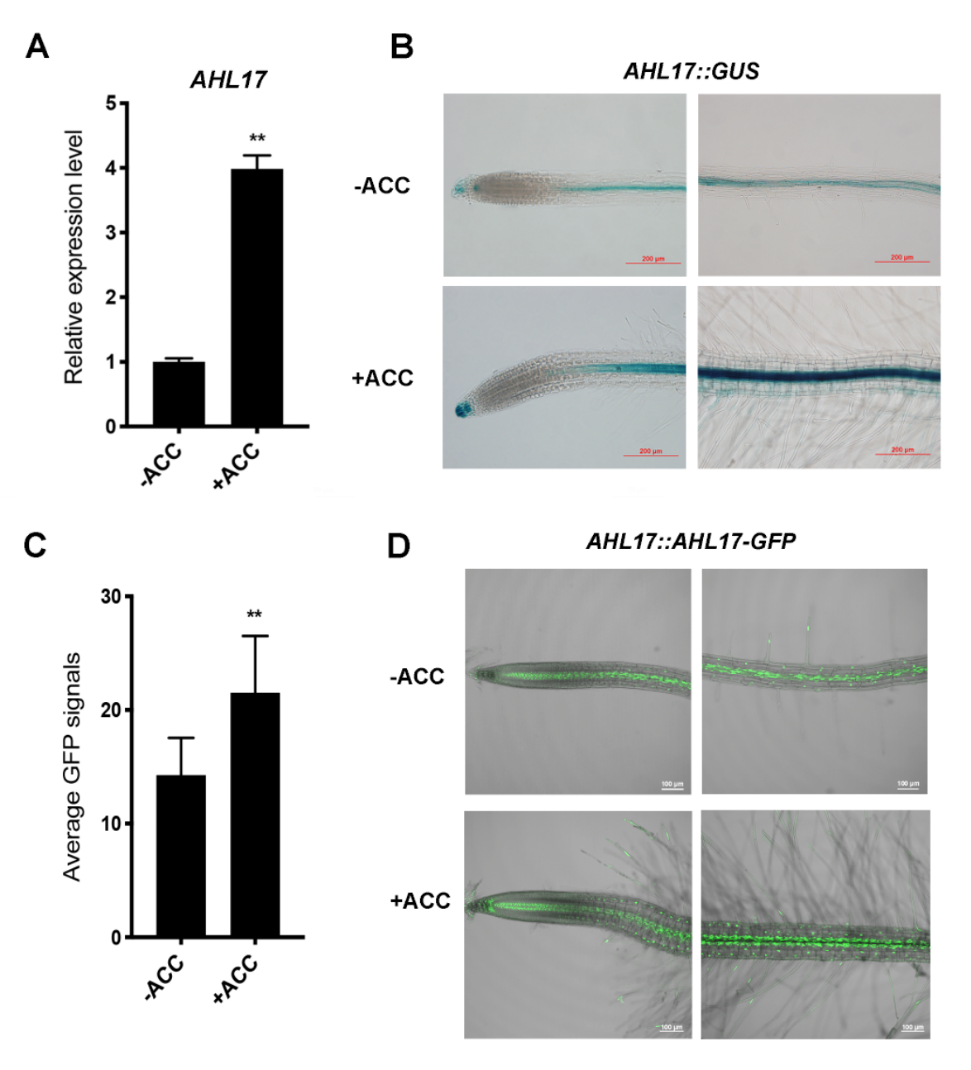


**Fig. S5 Effects of ACC treatment on gene transcription and protein accumuation of AHL17.**

(A) Relative expression of *AHL17* in 7-day-old seedlings of the WT grown on medium in absence (-) or presence (+) of 5 μM ACC as determined by RT-qPCR. Values are means ± SD of three biological replications. The expression of *AHL17* in the seedlings grown on medium without ACC was set to 1.0. Asterisks indicate a significant difference from the WT (*t*-test, **P < 0.01). (B) Histochemical staining of 7-day-old *AHL17::GUS* seedlings grown in the absence or presence of ACC. (C) Quantification of GFP fluorescence signals in seedlings shown in (D). Values are means ± SD of 15 roots of ACC-treated or non-treated seedlings, respectively. Asterisks indicate a significant difference from the WT (*t*-test, **P < 0.01). (D) Confocal microscopy images showing the accumulation of GFP-AHL17 proteins in 7-d-old seedlings in absence or presence of 5 μM ACC.

**Supplemental Figure S6**


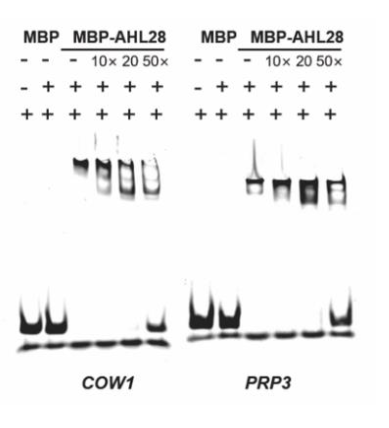

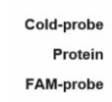


# Fig. S6 AHL28 binds to the promoters of the *COW1* and PRP3 genes.

EMSAs showing the binding of AHL28 to the putative AT-hook binding elements in the promoters of the *COW1* and *PRP3* genes. The experiment was performed using 1.5 μg MBP-AHL28 and 3 μg MBP proteins. The working concentration FAM-labelled probe was 1μM. Different amounts of excess unlabeled probe (cold probe) were added as competitors.

**Supplemental Figure S7**

**
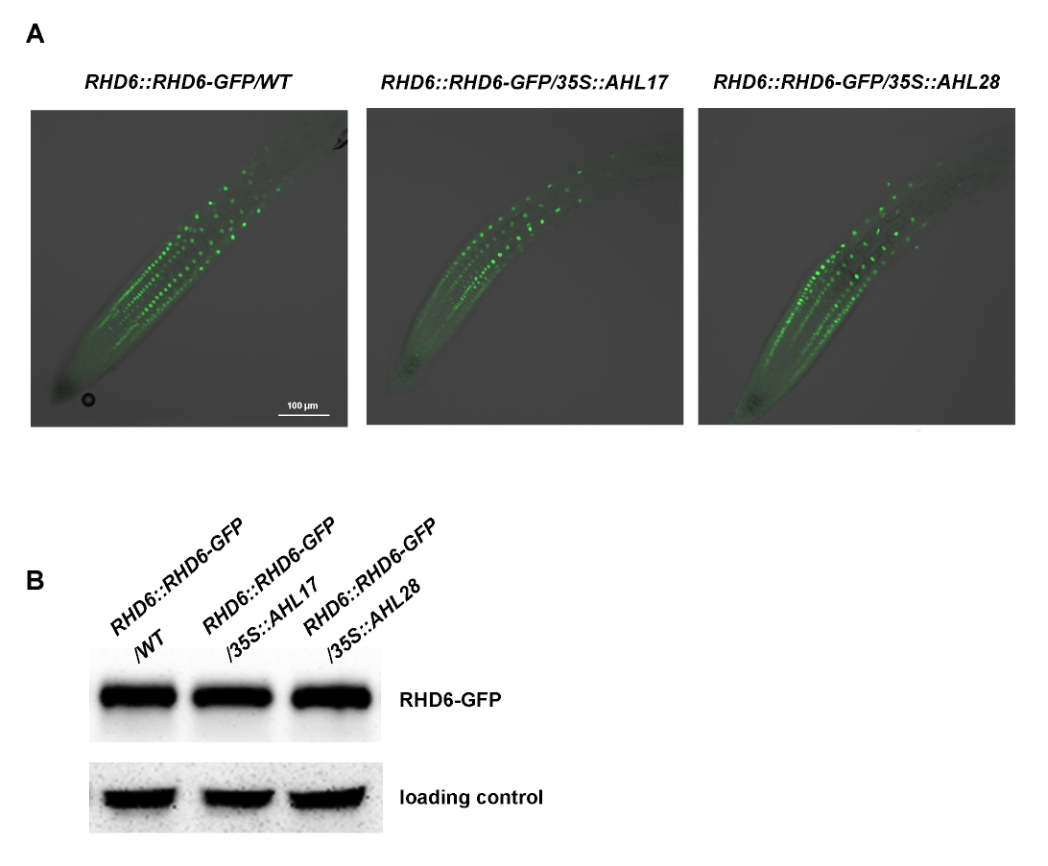
**

# Fig. S7 Accumulation of RHD6-GFP fusion proteins in the WT, *35S::AHL17,* and *35S::AHL28* transgenic plants.

# (A) Confocal microscopy images of the root tips of 7-day-old seedlings of F_1_ progeny derived from the cross between *RHD6::RHD6-GFP* and WT, *35S::AHL17*, or *35S::AHL28* lines. (B) Western blot analysis of RHD6 protein accumulation in the roots of F_1_ plants derived from cross between *RHD6::RHD6-GFP* and WT, *35S::AHL17*, or *35S::AHL28* lines. The total proteins were extracted from roots of 7-d-old seedlings and RHD6-GFP were detected by Western blot analysis with anti-GFP antibody. Actin was used as a loading control.

**Supplemental Figure S8**

**
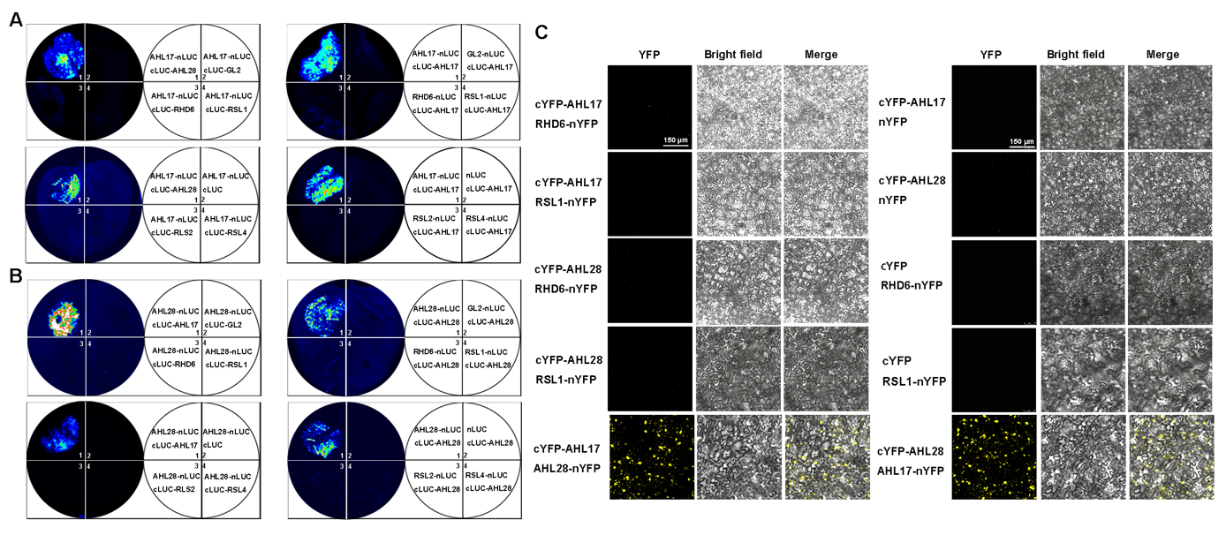
**

# Fig. S8 Interaction between AHL17/AHL28 and major root transcription factors involved in root hair development.

# (A) and (B) LIC assays. A pair of constructs with various combinations (indicated in the diagrams on right) were co-transformed into the leaves of *N. benthamiana.* LUC activity was detected 2 d after infiltration to determine the interaction between various transcription factors with AHL17 (A) or AHL28 (B). The co-transformation of *AHL17-nLUC* and *cLUC-AHL28*, *cLUC-AHL17* and *AHL28-nLUC*, *AHL17-nLUC* and *cLUC-AHL17*, or *AHL28-nLUC* and *cLUC-AHL28* was used as a positive control. (C) BiFC assays. A pair of constructs of various combinations were co-transformed into the leaves of *N. benthamiana.* Yellow fluorescence signals were detected 2 d after infiltration to determine the interaction between AHL17 and AHL28 with RHD6 and RSL1, respectively. The co-transformation of *cYFP-AHL17* and *AHL28-nYFP* or *cYFP-AHL28* and *AHL17-nYFP* was used as the positive control.

**Supplemental Figure S9**


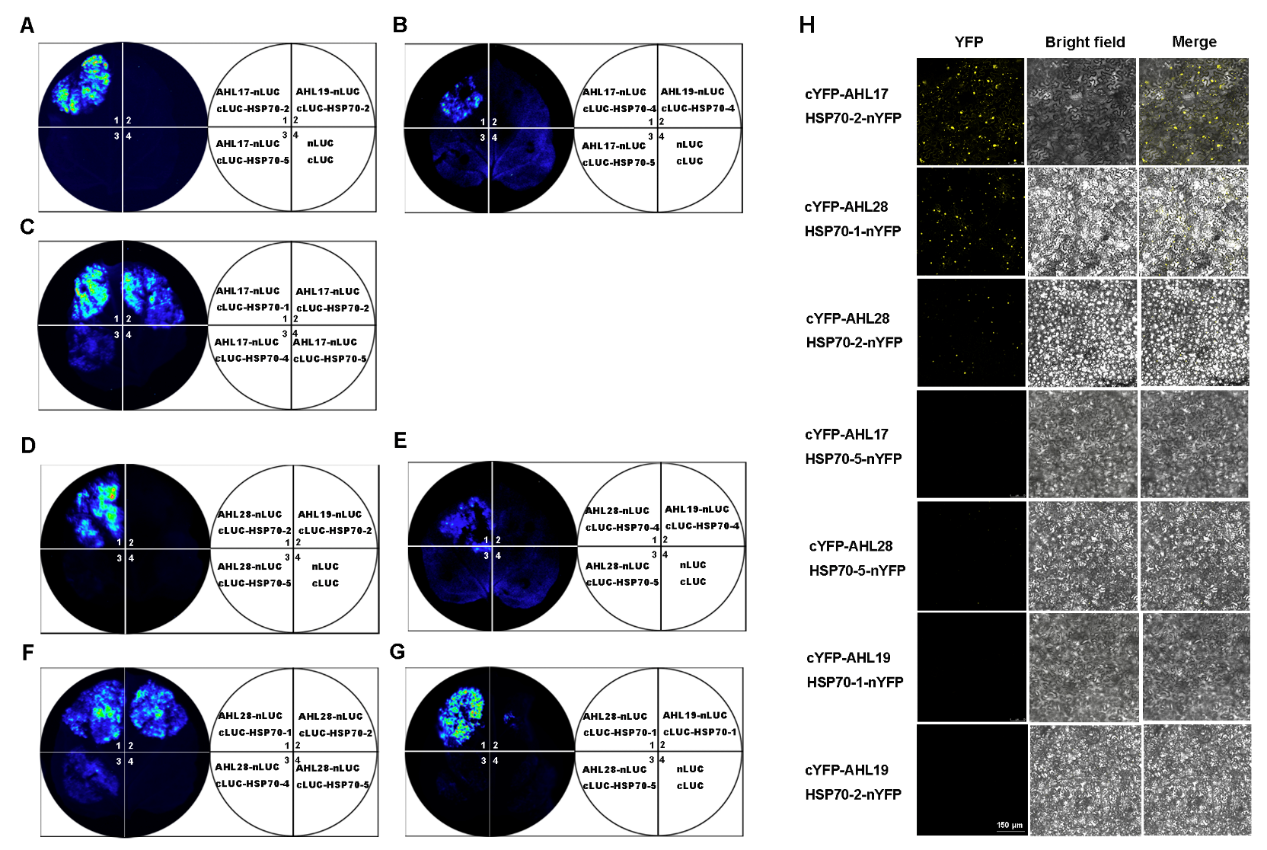


**Fig. S9 Interaction between AHL17/AHL28 and three HSP70 proteins.**

(A) to (C) LIC assays. A pair of constructs with various combinations (indicated in the diagrams on right) were co-infiltrated into the leaves of *Nicotiana benthamiana* to examine the interactions between AHL17 with three HSP70 proteins. Luciferase activity was detected 2 d after infiltration. (A) and (B) Interactions between AHL17 and HSP70-2 and HSP70-4. (C) Relative strength of the interactions between AHL17 with HSP70-1, HSP70-2, HSP70-4, and HSP70-5. (D) to (G) LCI assays. The interactions between AHL28 and HSP70-2 (D), AHL28 and HSP70-4 (E). (F) Comparison of the relative strength of the interactions between AHL28 with HSP70-1, HSP70-2, HSP70-4, and HSP70-5. (G) Specific interactions between AHL28 and HSP70-1. (H) Specific interactions between AHL17 and HSP70-2, between AHL28 and HSP70-1, and between AHL28 and HSP70-2 as indicated by BiFC assays in *N. benthamiana*.

**Supplemental Figure S10**


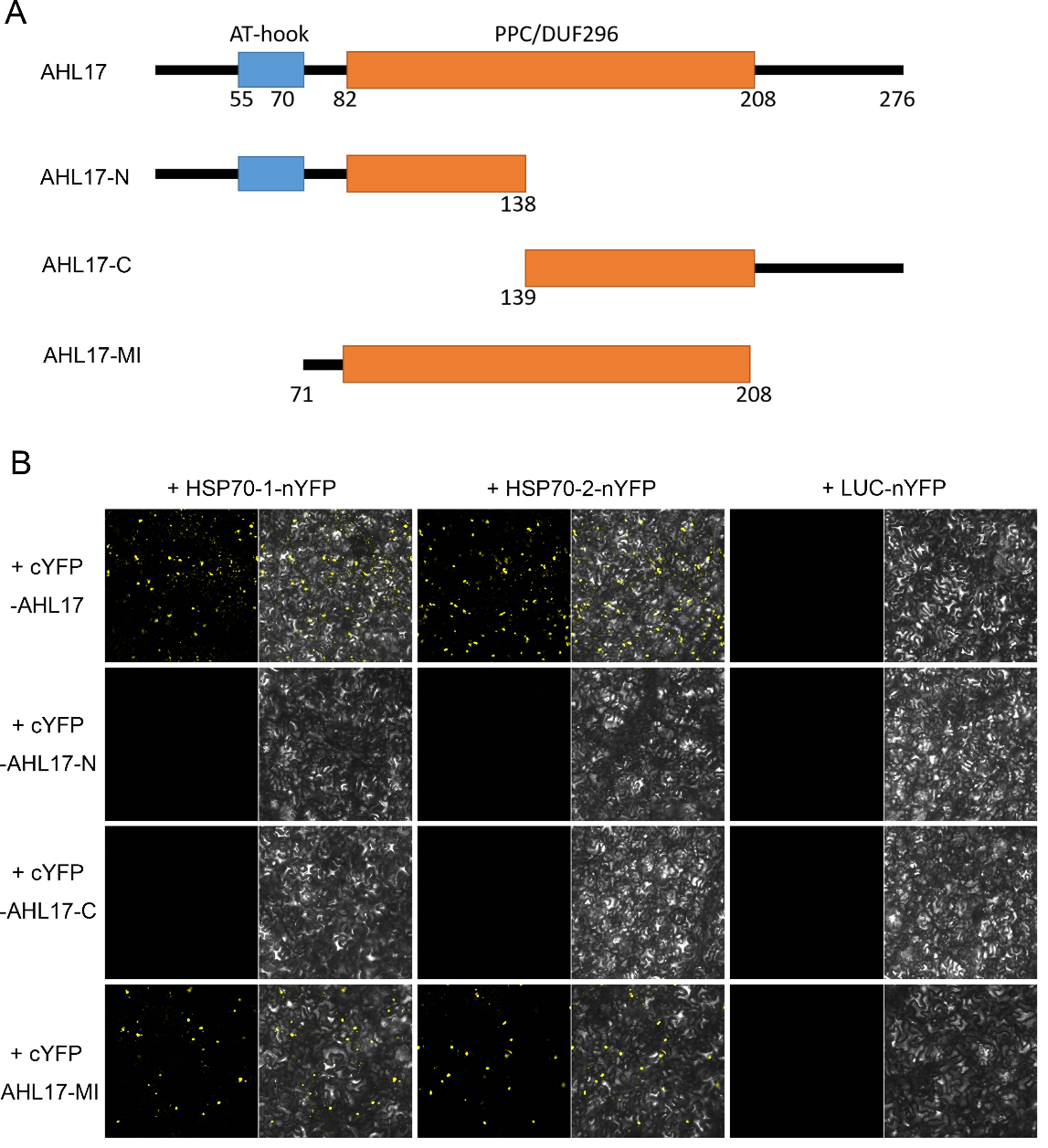


# Fig. S10 The protein domain of AHL17 involved in the interactions between AHL17 and HSP70-1 and HSP70-2.

# (A) Diagrams showing different parts of AHL17 that were used in BiFC assays. (B) BiFC assays. A pair of constructs of various combinations (as indicated on the left and top of the panel) were co-transformed into the leaves of *N. benthamiana.* Yellow fluorescence signals were detected 2 d after infiltration to determine the interaction between different parts of AHL17 and HSP70-1 and HSP70-2, respectively. The co-transformation of LUC-nYFP with cYFP-AHL17 was used as the negative control.

**Supplemental Figure S11**


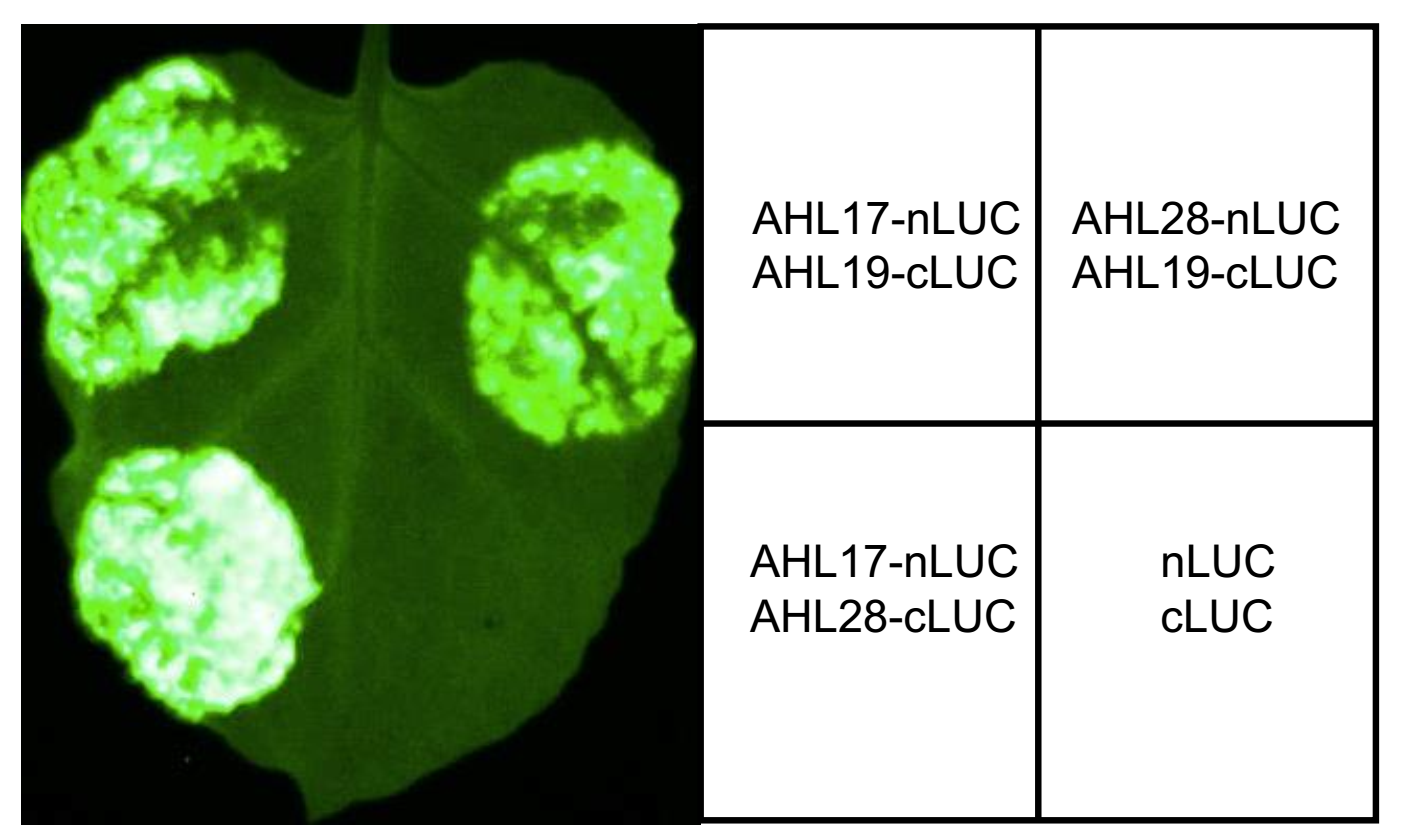


**Fig. S11 Interactions between AHL17/AHL28 and AHL19.**

LCI assays were used to test the interaction between AHL17/AHL28 and AHL19. The CDS of AHL17 and AHL28 were fused to the N-terminal half (nLUC) of the LUC gene and the CDS of AHL19 was fused with the C-terminal half (cLUC) of the LUC gene. A pair of constructs with various combinations (indicated in the diagrams on right) were co-infiltrated into the leaves of *Nicotiana benthamiana* to examine the interactions between AHL17/AHL28 and AHL19. Luciferase activity was detected 2 d after infiltration. The co-infiltration of the constructs of AHL17-nLUC and AHL28-cLUC was used as a positive control and co-infiltration of the constructs nLUC and cLUC was used as a negative control.
